# Human amniotic mesenchymal stromal cell-derived extracellular vesicles reprogram microglia and prevent neurodegeneration in experimental models of Alzheimer’s disease

**DOI:** 10.1186/s40035-026-00581-1

**Published:** 2026-09-09

**Authors:** Andrea Papait, Francesca Natale, Antonietta Rosa Silini, Ida Nifo Sarrapochiello, Elisa Orecchini, Raimondo Sollazzo, Marco Rinaudo, Serafina Farigu, Nicoletta Garofalo, Giulia Mantini, Guido Maria Giuffrè, Pietro Romele, Elvira Ragozzino, Alice Dellaria, Luciano Giacò, Camillo Marra, Cristian Ripoli, Claudio Grassi, Ornella Parolini, Salvatore Fusco

**Affiliations:** 1 Dipartimento di Scienze della Vita e Sanità Pubblica, Università Cattolica del Sacro Cuore, 00168 Rome, Italy; 2 Fondazione Policlinico Universitario A. Gemelli IRCCS, 00168 Rome, Italy; 3 Dipartimento di Neuroscienze, Università Cattolica del Sacro Cuore, 00168 Rome, Italy; 4 Centro di Ricerca E. Menni, Fondazione Poliambulanza Istituto Ospedaliero, 25124 Brescia, Italy; 5 UOS Computational Biology and Bioinformatics GSTeP, Fondazione Policlinico Universitario A. Gemelli IRCCS, 00168 Rome, Italy; 6 Dipartimento di Psicologia, Università Cattolica del Sacro Cuore, 20123 Milano, Italy; 7 Fondazione IRCCS Casa Sollievo Della Sofferenza, 71013 San Giovanni Rotondo, Foggia Italy

**Keywords:** Extracellular vesicles, Microglia, iPSC-derived human neurons, Neuroinflammation, Neuroplasticity

## Abstract

**Background:**

Alzheimer’s disease (AD) is the most prevalent neurodegenerative disorder and disproportionately affects women, with neuroinflammation emerging as a key driver of disease onset and progression. Beyond amyloid-β (Aβ) and hyperphosphorylated tau protein accumulation, chronic activation of microglia and astrocytes amplifies synaptic dysfunction and neuronal loss. Mesenchymal stromal cell-derived extracellular vesicles (MSC-EVs) represent a promising translational strategy due to their capacity to modulate inflammation and promote neuroprotection. Here, we investigated whether intranasal administration of extracellular vesicles derived from human amniotic membrane MSCs (hAMSC-EVs) could counteract cognitive decline, neuroinflammation, and synaptic alterations in experimental and human cellular models of AD.

**Methods:**

hAMSC-EVs were isolated and characterized for size, markers, and biodistribution. Female 3 × Tg-AD mice received chronic intranasal hAMSC-EV administration from 3 to 9 months of age. Cognitive performance was assessed using novel object recognition, object place recognition, and Y-maze tests. Hippocampal Aβ levels, tau phosphorylation, glial density, microglial morphology, cytokine profiles, and synaptic protein expression were analyzed by immunoblotting, ELISA, immunofluorescence, and morphometric analyses. Bioinformatic analyses were performed to investigate the miRNA cargoes of hAMSC-EVs. Translational relevance of the hAMSC-EV effects was assessed in glutamatergic neurons differentiated from induced pluripotent stem cells derived from sporadic AD patients.

**Results:**

The hAMSC-EVs delivered intranasally reached the hippocampus and were internalized by neurons and microglia. hAMSC-EV treatment significantly improved cognitive performance of female 3 × Tg-AD mice and reduced hippocampal Aβ levels without affecting tau phosphorylation. The hAMSC-EVs attenuated neuroinflammation by reducing microglial and astrocytic density, inducing microglial structural remodeling, and downregulating TMEM119 and TREM2 expression. We also detected a shift toward an anti-inflammatory cytokine profile and increased expression of neuroplasticity-related proteins, including BDNF, GluA1, and ARC in the hippocampus of 3 × Tg-AD mice. Bioinformatic analyses identified EV miRNA cargoes enriched in immunomodulatory and neuroprotective pathways. In human AD neurons, hAMSC-EVs prevented neurite atrophy and rescued synaptic protein expression without affecting the cell viability.

**Conclusions:**

hAMSC-EVs exert robust anti-inflammatory and neuroprotective effects in both murine and human AD models, improving cognition, modulating glial activation, and restoring synaptic integrity. These findings highlight the translational potential of intranasal hAMSC-EVs as an adjuvant therapeutic strategy targeting neuroinflammation and neurodegeneration in AD.

**Supplementary Information:**

The online version contains supplementary material available at https://doi.org/10.1186/s40035-026-00581-1.

## Background

Alzheimer’s disease (AD) stands as the most prevalent form of neurodegenerative disorder, characterized by progressive and irreversible loss of neurons, predominantly affecting critical brain regions such as the hippocampus and cortex [1, 2]. This pathological condition initially manifests as memory impairment, subsequently progressing to impact other essential cognitive functions, severely compromising daily life [3, 4]. AD poses a huge global public health challenge, with its incidence escalating exponentially after age 65. Notably, epidemiological data consistently highlight a higher prevalence among women [4, 5].

The core neuropathological features of AD include accumulation of amyloid-β (Aβ) peptides into extracellular oligomers and formation of intracellular neurofibrillary tangles (NFTs) due to the hyperphosphorylation of tau protein [6]. This aggregation occurs alongside a pronounced state of cerebral atrophy, resulting from the massive loss of neurons and synapses [7]. This neurodegeneration is exacerbated by neuropathological conditions like oxidative stress and cholinergic neuronal damage [8, 9].

A further important factor significantly contributing to AD pathogenesis is the dysregulation of local inflammatory milieu that occurs across various neurodegenerative disorders such as Parkinson’s disease and multiple sclerosis [10]. Crucially, cerebral neuroinflammation is emerging as an active pathogenic factor, not a mere consequence of cell death but an active contributor to both the onset and the chronic progression of AD [11]. This complex, local innate immune response involves the crucial interplay of different glial cells such as microglia and astrocytes, driving the release of various pro-inflammatory cytokines (e.g., IL-1β, IL-6, TNF-α), exacerbating AD neuropathological hallmarks and severely compromising neuronal survival [12, 13]. The complex and multifactorial nature of AD poses substantial challenges for developing truly effective therapeutic interventions, with current strategies aiming either to manage symptoms or modify the underlying disease progression. Given the strong evidence for the inflammatory role, effective management of associated chronic inflammatory conditions represents a highly promising and targeted approach for prevention, risk reduction, and development of novel therapeutic strategies aimed at mitigating disease progression. Recent efforts intensely focused on identifying targets that address these core pathological mechanisms [11, 14]. A particularly promising avenue within regenerative medicine involves harnessing the therapeutic potential of extracellular vesicles (EVs), particularly those isolated from mesenchymal stromal cells (MSCs) [15, 16]. MSC-derived EVs (MSC-EVs) are nanoparticles that mediate intercellular communication and have been demonstrated to potentially deliver bioactive molecules with anti-inflammatory and neuroprotective properties [17–19]. In this study, we investigated whether chronic intranasal administration of human amniotic mesenchymal stromal cell (hAMSC)-EVs could mitigate cognitive decline and neuropathological alterations in female 3 × Tg-AD mice. We combined behavioral, molecular, histological, and bioinformatic analyses to assess the effects of hAMSC-EVs on neuroinflammation, synaptic plasticity, and glial function. Finally, we evaluated the translational relevance of this approach using induced pluripotent stem cell (iPSC)-derived glutamatergic neurons generated from patients with sporadic AD (sAD).

## Materials and methods

### Isolation of hAMSCs and collection of conditioned medium (CM)

hAMSCs were isolated from term placental amniotic membranes as described previously [20]. Briefly, membranes were minced and initially digested at 37 °C for 9 min with 2.5 U/mL dispase (VWR, Radnor, PA). The enzymatic reaction was halted by washing the tissue fragments in RPMI 1640 medium supplemented with 10% heat-inactivated fetal bovine serum (FBS), 1% penicillin/streptomycin, and 1% L-glutamine (all from Sigma-Aldrich, St. Louis, MO). A second enzymatic digestion was performed using 0.94 mg/mL collagenase and DNase I (Roche, Basel, Switzerland) for 2.5–3 h at 37 °C. The resulting cell suspension was filtered through a 100-μm cell strainer (BD Falcon, Bedford, MA), followed by low-speed centrifugation to collect the cells.

Cells at passage 0 (p0) were characterized phenotypically according to established criteria [20]. Only preparations with > 80% expression of CD13 and CD90 and <10% expression of CD45 and CD324 were used. Cells were expanded to passage 1 (p1) in Chang Medium C (Irvine Scientific, Santa Ana, CA) supplemented with 2 mM L-glutamine, incubated at 37 °C with 5% CO_2_.

For CM production, p1 hAMSCs were seeded at a density of 5 × 10^5^ cells per well in 24-well plates (Corning, Corning, NY) and cultured for 5 days in 0.5 mL of serum-free DMEM/F12 (Sigma-Aldrich) supplemented with 2 mM L-glutamine and 1% penicillin/streptomycin, as described previously [20]. At the end of the culture period, CM was collected, centrifuged at 300× *g*, and filtered through a 0.2-μm sterile filter (Sartorius Stedim, Florence, Italy). A 5-mL aliquot of CM was stored at −80 °C for subsequent use as whole CM, while the remaining volume (15–20 mL) was used for EV isolation.

A total of six independent hAMSC-CM preparations were generated from six different placental donors.

### Animals and treatments

Female 3 × Tg-AD mice (B6;129-Tg(APPSwe,tauP301L)1Lfa Psen1tm1Mpm/Mmjax) were obtained from the Animal Facility of Università Cattolica del Sacro Cuore (Rome, Italy). Mice were housed in groups (3–5 animals per cage) under a 12-h light–dark cycle at constant temperature (22–24 °C) with food and water *ad libitum*. All studies were developed in compliance with the ARRIVE guidelines. Animals were randomly allocated to experimental groups using a computer-generated random sequence. The randomization method and allocation procedure were predefined in the study protocol to minimize selection bias. Factors such as litter origin, age, and body weight were considered during allocation to ensure comparable groups. Outcome assessment and data analysis were performed blinded to group assignment where possible. Mice derived from the same litter were randomly assigned to all the following experimental groups. 3 × Tg-AD mice were intranasally injected with (1) hAMSC-EVs (approximately 1 × 10^8^ diluted in 4 μL of 1 × phosphate-buffered saline (PBS) per administration) or (2) saline, starting from 3 months, a stage at which female 3 × Tg-AD mice still do not show any cognitive impairment, till 9 months of age, twice a week. The number of vesicles for injection was determined according to our previous studies [21, 22]. For a subset of experiments shown in Fig. S1, intranasal administration was carried out for one month starting at 9 months of age. The daily dose and frequency of treatment were similar to the standard protocol of administration.

For EV isolation, the media from hAMSCs were collected and pre-cleared using a multistep centrifugation protocol. EVs were purified using the exoEasy Maxi Kit (Qiagen, #76064,  Limburg, Netherlands) according to the manufacturer’s instructions. Isolated EVs were subjected to PBS buffer exchange using Vivaspin 2 columns (Sartorius, #VS0232, Goettingen, Germany) for in vivo treatment. Once isolated, the EVs were stored at −80 °C until use.

### EV characterization

EVs were analyzed and quantified by nanoparticle tracking analysis (NTA) using a Nanosight NS300 particle size analyzer (Malvern Panalytical, Malvern, UK). As previously described [22], for transmission electron microscopy (TEM) analyses, EVs were fixed with formaldehyde and glutaraldehyde in 0.1 mol/L sodium cacodylate buffer (pH 7.4), placed onto Formvar-carbon-coated grids and air-dried for 10 min. After being rinsed, vesicles were postfixed in 1.5% osmium tetroxide in 0.1 mol/L cacodylate buffer (pH 7.3) and then allowed to dry. Samples were observed with a Zeiss Libra 120 (Zeiss NTSGmbH, Oberkochen, Germany). Once isolated, the EV were stored at −80 °C until use.

### Microglial primary cultures

Primary microglial cells were isolated using the differential binding method, which separates primary astrocytes, oligodendrocyte progenitor cells and microglial cells based on their different affinities to bacterial grade plastic dishes [23]. Briefly, brains of mouse pups were dissected using dissection tools, a microscope, a cold light source and 70% ethanol under the dissection hood. After obtaining meninges-free brains, hippocampal tissues were isolated in 1 × HBSS (Gibco, Thermo Fisher Scientific Group, Waltham, MA). Once dissociated after incubation with DNAse I and Trypsin (Merck, Darmstadt, Germany), cells were plated in DMEM supplemented with 10% FBS and 2% pen/strep. After 10 days in vitro (DIV), microglial cells and astrocytes were parted, and almost pure microglial cell cultures were plated for 10 days before the start of the experiment.

### Microglial phagocytosis assay

Primary cultures of microglia were seeded and treated with (1) 1 µM Aβ fluorescent peptide alone for 6 h (CTRL), (2) 100 ng/µL lipopolysaccharide (LPS) and Aβ fluorescent peptide for 6 h (LPS), or (3) 6 × 10^5^ EVs (calculated as 200 EVs per cell) for 24 h followed by LPS and Aβ fluorescent peptide for 6 h, in the dark. To assess the phagocytic rate, cells were fixed for 15 min with 4% paraformaldehyde. After Alexa Fluor™ 546 F-actin (1:500 for 45 min; Invitrogen, Thermo Fisher Scientific Group, Waltham, MA) and 4′,6-diamidino-2-phenylindole (DAPI; 0.5 µg/mL for 10 min; Invitrogen) staining, cells were coverslipped with ProLong Gold anti-fade reagent (Invitrogen). Images (1024 × 1024 pixels) were acquired at 20 ×  and 40 × magnification with a Nikon A1 MP confocal system (Tokyo, Japan) and a non-oil-immersion objective (NA 1.2). The levels of phagocytosed peptide and microglial overactivation were evaluated either through green fluorescence intensity/blue fluorescence intensity, or by manually counting multinucleated cells/the total number of cells, in a defined region of interest (ROI). Each ROI was sized to 1 mm^2^.

For Western blot experiments, primary cells were treated with 100 ng/mL LPS for 24 h. A group of LPS-treated primary cells were pretreated with EVs at a dose of 200 EVs/cell 12 h before the LPS-treatment, to assess the effect of EV treatment on LPS-derived inflammation.

### Behavioural tests

All behavioural tests were performed as previously described [22]. Long-term recognition memory and short-term spatial memory were evaluated using novel object recognition (NOR) and object place recognition (OPR) tests, respectively. The Y-maze test was used to assess spatial working memory. In the NOR test, animals were familiarized for 10 min with the test arena (45 cm × 45 cm) on the first day (habituation). On the second day (training session), the mice were allowed to explore two identical objects placed symmetrically at the center of the arena for 10 min. On the last day (test session), one of the old objects was replaced with a new one, and the animals were allowed to explore for 10 min. In the OPR test, animals underwent the same procedure as the NOR test, except that during the test phase, one of the objects was moved into a different position without being substituted. In the OPR test, different cues were placed on the walls of the testing arena to provide spatial reference. Mice showing a total exploration time less than 10 s or spending significantly different time between the two objects during the training phase were excluded. Different couples of objects were employed between NOR and OPR. Preference index was calculated in the test phase as the ratio of the time spent exploring the novel object to the total time of object exploration to quantify recognition and spatial memory.

For Y-maze, a three-armed apparatus (40 cm × 9 cm × 15 cm, length, width, height) was used with each arm positioned at a 120° angle. Animals were habituated to the testing room for 30 min before the trial. Each animal was then placed in the starting arm and allowed to freely explore for 8 min. The percentage of spontaneous alternation was calculated as the ratio of the number of correct triplets to the total number of arm entries minus 2.

Tests were video-recorded, and analysis was performed in a blinded manner. To prevent the influence of odor cues on animal behavior, the maze and the objects in NOR and OPR were thoroughly cleaned with a 70% ethanol solution between tests.

### Western blot

Samples for western blot analyses were processed as previously described [24, 25]. Briefly, whole hippocampi were lysed in ice-cold lysis buffer (NaCl 150 mM, Tris–HCl 50 mM pH 8, EDTA 2 mM) containing 1% Triton X-100, 0.5% SDS, 1 × protease inhibitor cocktail, 1 mM sodium orthovanadate, 1 mM sodium fluoride and 1 mM phenylmethyl sulfonyl fluoride (all reagents from Sigma Aldrich). The lysate was sonicated with a Diagenode Bioruptor Standard water bath sonicator and incubated 15 min on ice. At the end of the incubation, samples were centrifuged at 13,000 ×  *g* at 4 °C for 15 min. The supernatant was quantified for protein content using Bradford assay (DC Protein Assay; Bio-Rad, Hercules, CA). Equal amounts of proteins were diluted in 6 ×  Laemmli Buffer, boiled, and resolved using sodium dodecyl-sulfate polyacrylamide gel electrophoresis (SDS-PAGE).

Human neurons derived from human induced pluripotent stem cells (hiPSCs) from sAD patients (sAD-hNs) and healthy subjects (HS-hNs) cultured in 6-well plates were lysed as previously described [24]. Cells underwent two PBS washes and were subsequently lysed in cold RIPA buffer containing 1 mM phenylmethylsulfonyl fluoride, phosphatase and protease inhibitor mixtures (#52332, Sigma-Aldrich), and 0.1% SDS. Following a 30-min incubation on ice, the cellular suspensions were centrifuged at 10,000 ×  *g* for 30 min at 4 °C. The resulting supernatants were collected and assessed for protein concentration using the Micro BCA protein assay (Thermo Fisher Scientific, #23235, Waltham, MA). Subsequently, the proteins were electroblotted onto nitrocellulose membranes. The membranes were blocked with 5% nonfat dry milk in Tris-buffered saline containing 0.1% Tween-20 (#P1379, Sigma-Aldrich) for 1 h at room temperature, and then incubated overnight with primary antibodies (Table S1) at a final concentration of 1:1000 in 3% milk in TBS-Tween, and then revealed with HRP-conjugated secondary antibodies (1:5000, # 7074 and # 7076, Cell Signaling Technology Inc. Danvers, MA) and different chemiluminescent substrates (#XLS1420250, XLS0750100, or XLS30100, Cyanagen, BO, Italy), according to the expected protein concentrations.

Signals were acquired on a UVItec Cambridge Alliance instrument and quantified with Alliance Software. The expression levels of the target proteins were normalized to the total amount of housekeeping protein (HSP90, β-actin or β-tubulin). The phosphorylation levels of the target proteins were normalized to the total amount of the target protein in each lane and to the total amount of housekeeping protein according to the following formula (phospho-protein/total protein/housekeeping protein). In bar graphs, where relative units were used to show the data, the mean value of the control was set to 1. Representative images of western blot were cropped for presentation with no manipulations. Uncropped blot images are provided in Fig. S4.

### Immunofluorescence analyses

For all the experiments, 3 × Tg-AD mice were overdosed with a mixture of ketamine and medetomidine (150 mg/kg and 1.5 mg/kg) administered through intraperitoneal injection, and transcardially perfused with PBS (0.1 M, pH 7.4), followed by fixation with 4% paraformaldehyde. Brains were post-fixed overnight at 4 °C in the same fixative, cryoprotected in a solution of 1× PBS containing 30% sucrose for 48–72 h, embedded in optimal cutting temperature (OCT) compound, and coronally sectioned at a thickness of 30 μm using a cryostat (SLEE, Mainz, Germany).

### EV labelling and biodistribution fluorescence analysis

For in vivo EV visualization, EVs were labeled with the ExoGlowTM Membrane EV Labeling Kit (System Biosciences, #EXOGM600A-1, Palo Alto, CA). Briefly, the labeling reaction buffer (composed by 12 µL of Reaction Buffer and 2 µl of Labeling Dye) was mixed with either 1 × 10^10^ EVs in 1×PBS or with 1× PBS alone (negative control). After a brief incubation (30 min at room temperature [RT]), 1-min centrifugation was performed to remove unbound dye using a buffer exchange PD SpinTrap G-25 column (Cytiva, #28918004, Marlborough, MA). Moreover, we carried out a reprecipitation protocol with ExoQuick-TC (System Biosciences, #EXOTC10A-1) to further clean the sample before proceeding with downstream applications. To assess localization of labelled EVs in brain tissue, mice were intranasally treated with 4 μL PBS (2 µL per nostril) containing 1×10^9^ EVs (*n* = 3 mice) or negative control (*n* = 3 mice) and six hours later, the animals were deeply anesthetized, sacrificed and perfused as previously described. Coronal sections were protected from light and subsequently stored at −20 °C in anti-freeze buffer solution until use. Sections were processed as previously described [21]. After permeabilization and 1-h blocking in 1× PBS containing both 0.3% Triton X-100 (Sigma) and 5% NGS, tissues were incubated overnight at 4 °C with Iba-1 antibody (Immunological Sciences, AB-83747, 1:500, Rome, Italy) or Map2 (Cell Signaling Technology, #8707, 1:500) in blocking solution. The next day, sections were incubated for 90 min at RT with Alexa Fluor 488 anti-rabbit (Invitrogen, R415, 1:500). Finally, nuclei were counterstained with DAPI (0.5 μg/mL for 10 min; Invitrogen), and sections were coverslipped with ProLong Gold anti-fade reagent (Invitrogen). Images (1024 × 1024 pixels) were acquired at 4 × and 60 × magnification with a Nikon A1 MP confocal system (Tokyo, Japan) and a non-oil-immersion objective (NA 1.2). Using the NIS Elements AR 5.30.01 software and focusing on MAP2^+^/Exo-Glow^+^ or IBA-1^+^/Exo-Glow^+^ cells, we performed a stack-by-stack analysis, which allowed evaluating the fluorescence intensity along the 3 cross-sections XY, XZ, and YZ. Five slices were analyzed for each animal, and a total of four mice were included.

**Fig. 1:**
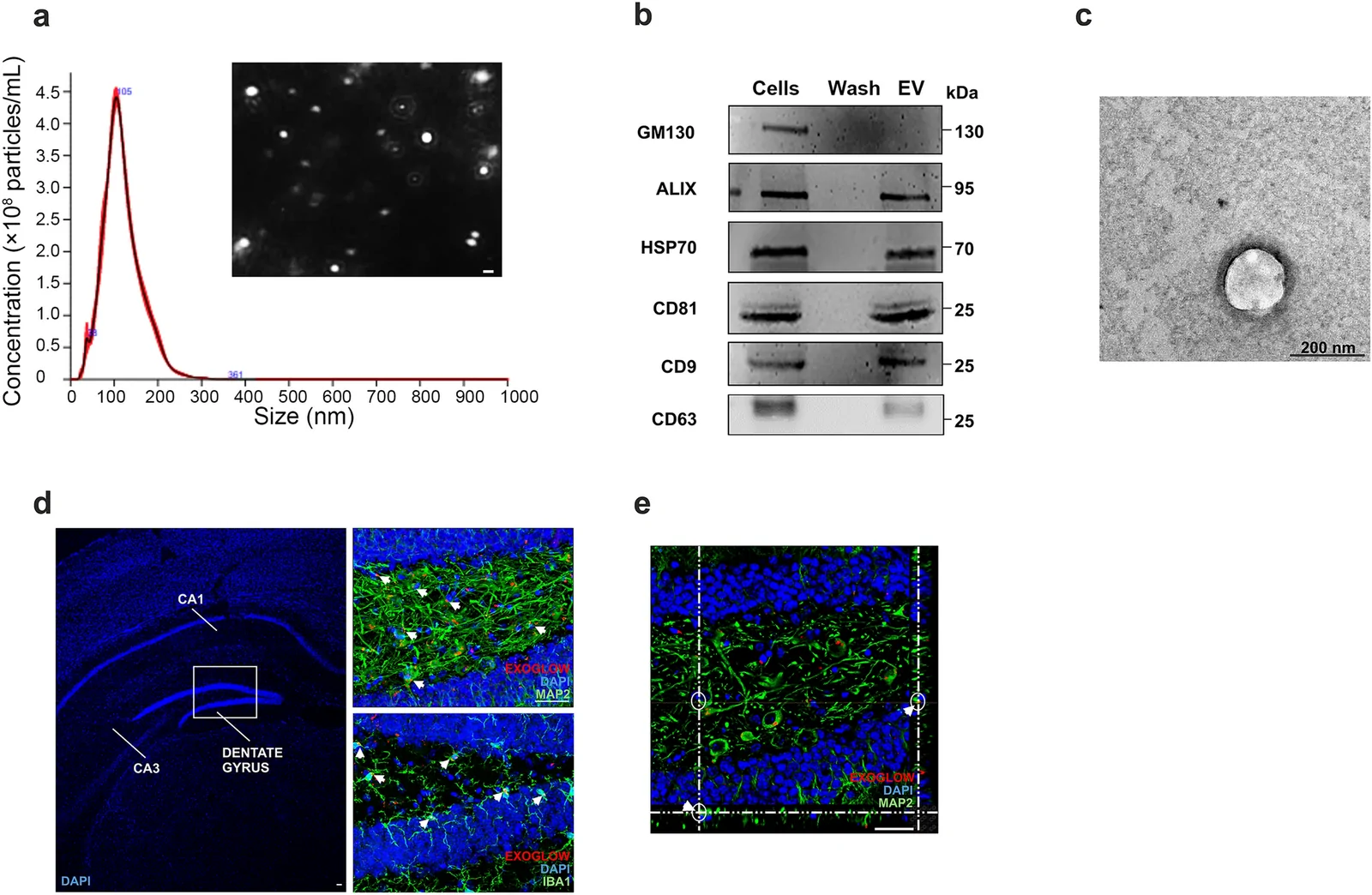
Characterization and biodistribution of hAMSC-EVs. **a** NanoTracking Analysis indicates the main size distribution (80–200 nm), and a representative image of purified hAMSC-EVs (top right). Scale bar: 100 nm. **b** Immunoblot analysis of EV markers Alix, HSP70, CD81, CD9, CD63, and the absence of GM130 (a marker of cellular contamination) in hAMSC-EV lysates. The “wash” lane represents the technical negative control. **c** A representative transmission electron microscopy image of EV isolated from hAMSC medium. **d** Representative images showing confocal staining in the hippocampus of mice intranasally treated with fluorescent hAMSC-EVs labelled with Exo-Glow™ (red). Arrows show aggregated EVs in neurons (MAP2^+^ cells, top right panel) and microglia (Iba1^+^ cells, bottom right panel). Scale bar: 50 µm. **e** A representative image of stack-by-stack analysis to evaluate the localization of Exo-Glow^TM^-stained EVs along the 3 cross-sections XY, XZ, and YZ. Experiments for EV characterization were performed at least three times. Scale bar: 50 µm

### Quantitative colocalization fluorescence analysis

Tissue sections were mounted onto Superfrost Ultra Plus Gold slides (Epredia, Thermo Fisher Scientific Group) and processed for immunofluorescence analysis. For Iba1 detection, antigen retrieval was performed using HIER Buffer L (1 × , pH 6; Thermo Fisher Scientific) at 98 °C for 5–10 min. Sections were incubated in blocking solution containing PBS 1 × , 0.3% Triton X-100 and 10% normal serum for 1 h at room temperature. Sections were then incubated overnight at 4 °C with the following primary antibodies: chicken anti-Iba1 (1:1000; RRID: AB_2942801, Invitrogen), chicken anti-GFAP (1:500; RRID: AB_10621124, GeneTex, Irvine, CA), rabbit anti-TMEM119 (1:250; RRID: AB_2887209, GeneTex), and goat anti-TREM2 (1:50; ab95470, Abcam).

After thorough washing, sections were incubated for 1.5 h at room temperature with species-specific secondary antibodies, including Alexa Fluor™ 568 goat anti-chicken IgY (A11041; Invitrogen), CY5-conjugated goat anti-rabbit IgG (CY-1500; Vector Laboratories), and DyLight 488-conjugated anti-goat IgG (DI-3088; Vector Laboratories). All secondary antibodies were used at a final concentration of 2 μg/mL–10 μg/mL.

Nuclei were counterstained with DAPI (0.5 µg/mL for 5 min; Invitrogen). Tissue sections were mounted using an VECTASHIELD® Vibrance™ Antifade Mounting Medium (H-1700; Vector Laboratories) and stored at 4 °C until imaging.

Image acquisition was conducted using the Mica Widefield Live Cell imaging platform (Leica Microsystems, Wetzlar, Germany). Considering the 30-μm section thickness, z-stacks were acquired with a standardized step size covering a total depth of 12 μm. To ensure consistency in fluorescence intensity across samples, all images were acquired using the Relight function. Four mice (with 4 slices per animal) were analyzed for each experimental group.

Fluorescence quantification was performed using ImageJ Fiji (NIH). Images were converted to 8-bit grayscale and subjected to standardized preprocessing, including background subtraction and thresholding using the "Default" method. ROIs were defined either manually or semi-automatically, and integrated density values for TMEM119⁺ and TREM2⁺ signals were calculated as the product of mean gray value and ROI area.

For Iba1⁺ and GFAP⁺ markers, positive cells were quantified using the Analyze Particles function following binary thresholding. Cell counts were normalized to tissue area and expressed as cells/mm^2^. Colocalization between markers was assessed using the Coloc 2 plugin in ImageJ, yielding Pearson’s correlation coefficients and colocalization maps.

All quantitative data were analyzed in a blinded manner using GraphPad Prism 9 (GraphPad Software, San Diego, CA) and reported as: (1) Iba1⁺ microglial cells per mm^2^, (2) GFAP⁺ astrocytes per mm^2^, (3) integrated density of TMEM119⁺ and TREM2⁺ signals, and (4) colocalization coefficients.

### Skeleton-based microglial morphometric analysis

To assess treatment-induced changes in microglial morphology, high-resolution confocal images of Iba1⁺ cells were obtained at 40 ×  magnification from selected brain regions. Images were converted to 8-bit grayscale and preprocessed with ImageJ filters (FFT Bandpass, Unsharp Mask, Close) to enhance structural features and suppress background noise. Binary images were then skeletonized using the Analyze Skeleton plugin (https://imagej.net/AnalyzeSkeleton). This analysis yielded objective metrics such as branch segment count, endpoint number, and junction complexity. Quantified parameters included the total process length, soma area, and soma perimeter. Analyses were performed on maximum intensity projections to optimize visualization of cellular morphology and were conducted in a blinded manner across experimental groups.

### ELISA

Aβ peptide 1–42 levels were analyzed on whole hippocampi using an Ultrasensitive Human Amyloid β 42 ELISA Kit (KHB3544, Thermo Fisher Scientific). Assays were performed as previously described [25] and according to the manufacturer’s instructions. The analysis was performed in a blinded manner.

### Cytokine analysis

Cytokine analysis in murine tissues was performed using the Q-Plex (15-Plex) Mouse HS Array™ Technology (112449MS-15P, Quansys Biosciences, Logan, UT). Q-Plex is an ELISA assay intended for simultaneous and quantitative measurements of multiple cytokines in a single sample: granulocyte–macrophage colony-stimulating factor (GM-CSF) (lower limit of quantification [LLOQ]: 0.61 pg/mL), interferon-gamma (IFNγ) (0.54 pg/mL), interleukin (IL)-1α (0.72 pg/mL), IL-1β (2.51 pg/mL), IL-2 (0.38 pg/mL), IL-3 (0.08 pg/mL), IL-4 (0.43 pg/mL), IL-5 (0.54 pg/mL), IL-6 (0.68 pg/mL), IL-10 (0.70 pg/mL), IL-12p70 (0.65 pg/mL), IL-17 (0.77 pg/mL), macrophage inflammatory protein (MIP)-1α (1.16 pg/mL), RANTES (regulated on activation, normal T cell expressed and secreted) (2.46 pg/mL), and tumor necrosis factor (TNF)-α (0.67 pg/mL).

Human cytokines were analysed by Q-Plex™ Human Cytokine Screen (16-Plex) (110933HU, Quansys Biosciences) according to the manufacturer’s instructions. Briefly, 50 µL of each sample (diluted 1:2) was added to each well. The multiplex ELISA kit contained in each well antibodies for detection of the following targets: IL-1α (LLOQ: 4.3 pg/mL), IL-1β (12.49 pg/mL), IL-2 (5.66 pg/mL), IL-4 (2.39 pg/mL), IL-5 (3.92 pg/mL), IL-6 (3.41 pg/mL), IL-8 (2.28 pg/mL), IL-10 (9.94 pg/mL), IL-12p70 (3.54 pg/mL), IL-13 (3.72 pg/mL), IL-15 (12.39 pg/mL), IL-17A (21.57 pg/mL), IL-23 (50.58 pg/mL), IFNγ (9.43 pg/mL), TNF-α (6.75 pg/mL), and TNF-β (4.86 pg/mL).

Each EV sample was analyzed in triplicate. A total of 2.5 × 10^9^ EVs were loaded into each well. Whole hippocampi or hAMSC-EVs were lysed and assay was performed according to the manufacturer’s instructions. Briefly, samples were pipetted into the wells of a 96-well plate with immobilized analyte-specific antibodies. After washing, a biotinylated analyte-specific antibody was added. After washing, streptavidin–horseradish peroxidase was added to the wells and incubated according to the manufacturer’s instructions. The amount of conjugated enzyme in each well was measured by adding a chemiluminescent substrate and results read using Q-View Software. The analysis was performed in a blinded manner. Statistical analysis was performed using multiple Student’s* t*-tests followed by Benjamini–Hochberg false discovery rate procedure.

### Depletion of vesicular RNA and miRNA analyses

For RNAse experiments, 5 × 10^9^ EVs were incubated with 6 µg of digitonin and 30 µg of RNAse enzyme (Invitrogen) (hAMSC-EV_R_) or digitonin alone (as control) at 37 °C for 2 h, with constant agitation. At the end of the incubation, EVs were isolated through ultracentrifugation, analyzed through NTA to verify membrane integrity, and resuspended in 1 ×  PBS for cell (microglia, iPSC) treatment, or in the resuspension buffer of the miRNeasy micro kit (#217084, Qiagen) for miRNA enrichment following manufacturer’s instructions. Once isolated, RNA was first analyzed through nanodrop reading and then prepared for reverse transcription with the miRCURY LNA RT Kit (#339340, Qiagen). 10 ng of total miRNA was retro-transcribed for each sample and analyzed by real-time PCR using the miRCURY LNA miRNA Focus PCR Panel Assay (YAHS-211YA, Qiagen) in 96-well plates according to the manufacturer’s instructions. All samples were analyzed in triplicate, and data were normalized to the levels of SNORD49A and UniSP3 (internal control) using the 2^−ΔΔCt^ method. Complete results are shown in Table S2.

### Fibroblast cultures

Skin punch biopsies (4 mm diameter; HBP40, HS, Italy) were collected from the perimalleolar region of three individuals diagnosed with sAD and three healthy subjects (HS). All participants provided written informed consent prior to enrollment, in compliance with Good Clinical Practice guidelines and local ethical regulations. Diagnosis of sAD was established according to the National Institute on Aging–Alzheimer’s Association criteria [26, 27], based on documented cognitive decline assessed through comprehensive neuropsychological testing and corroborated by AD biomarkers, including amyloid PET imaging or cerebrospinal fluid analyses. Age-matched neurotypical controls were recruited following informed consent procedures.

Biopsies were washed three times in Dulbecco’s phosphate-buffered saline (DPBS; #14040117, ThermoFisher Scientific) supplemented with 1% penicillin–streptomycin–neomycin (#15640055, ThermoFisher Scientific) and 1% gentamicin (#15710064, ThermoFisher Scientific) immediately after collection. Samples were then maintained in fibroblast growth medium consisting of high-glucose DMEM (#31053044, ThermoFisher Scientific) supplemented with 10% heat-inactivated FBS (#A3160501, ThermoFisher Scientific).

Under sterile conditions, tissue fragments were finely minced and plated onto 10 cm^2^ culture dishes precoated with 0.2% gelatin (G1393, Sigma-Aldrich) diluted in DPBS. To promote tissue attachment, fibroblast medium was added 8 h after plating. Cultures were maintained at 37 °C in a humidified atmosphere with 5% CO_2_, and the medium was replaced every 48 h until approximately 90% confluence was achieved. Fibroblasts were then enzymatically detached using TrypLE™ Express (1× ; #12605036, ThermoFisher Scientific) and expanded for subsequent reprogramming.

### Reprogramming of fibroblasts into hiPSCs

Dermal fibroblasts were converted into hiPSCs using a non-integrative Sendai virus-based approach (CytoTune™-iPS 2.0 Kit; #A16517, ThermoFisher Scientific), following the manufacturer’s instructions. Briefly, 5 × 10^5^ cells were seeded across two wells of a 6-well plate. Forty-eight hours later, fibroblasts cultured in standard growth medium were transduced using predefined multiplicities of infection (MOI): KOS (MOI = 5), hc-Myc (MOI = 5), and hKlf4 (MOI = 3). Twenty-four hours post-transduction, the viral medium was completely replaced with fresh fibroblast medium, which was subsequently refreshed every other day. On day 7, cells were detached, counted, and re-plated onto Matrigel-coated 6-well plates at densities ranging from 2 × 10^4^ to 1 × 10^5^ cells per well. After an additional 24 h, the culture medium was switched to StemFlex™ Medium (#A3349401, ThermoFisher Scientific) and renewed every 48 h. Emerging hiPSC colonies were observed between three and four weeks after transduction, manually isolated, and transferred to 12-well plates for further expansion and downstream analyses.

### hiPSC maintenance

Established hiPSC lines were maintained on Matrigel®-coated (#354277, Corning) 6-well plates in StemFlex™ Medium, with medium changes performed every other day. Cells were passaged upon reaching 70%–80% confluence using ReLeSR™ dissociation reagent (#100–0483, STEMCELL Technologies, Vancouver, Canada). Pluripotency was verified through immunocytochemical analyses (Fig. S2), as previously reported [24].

### Excitatory neuron differentiation

Excitatory neuronal differentiation was achieved through forced expression of Neurogenin-2 (NGN2) as previously described [24]. At the start of the differentiation procedure (day 0), hiPSC lines derived from sAD patients and healthy subjects (passage 10) were seeded at a density of 60,000 cells/cm^2^. Cells were plated in 6-well plates for protein extraction and western blot analyses, and in 24-well plates for immunocytochemistry and Incucyte®-based assays. Cultures were maintained in StemFlex™ Medium supplemented with the ROCK inhibitor Y-27632 (10 μM; #Y0503, Sigma-Aldrich). On day 1, cells were co-transduced with two lentiviral vectors (1 μL per well in 24-well plates): (i) TetO-hNGN2-eGFP-Puro, in which NGN2-eGFP-Puro expression is driven by a tetracycline-responsive TRE3G promoter, and (ii) FUW-M2rtTA, encoding the reverse tetracycline-controlled transactivator Tet3G, which enables doxycycline-dependent activation of the TRE3G promoter. Viral transduction was performed in StemFlex™ Medium containing Y-27632 (10 μM) and Polybrene (8 μg/mL; #TR-1003, Sigma-Aldrich). Plates were incubated for 10 min and subsequently centrifuged at 1000 ×  *g* for 1 h at RT to enhance infection efficiency, followed by overnight incubation at 37 °C. Twenty-four hours after transduction, the culture medium was fully replaced with fresh StemFlex™ Medium supplemented with doxycycline (2 μg/mL; #D9891, Sigma-Aldrich). Doxycycline was maintained throughout the entire differentiation period to sustain NGN2 expression. On day 3, cells were switched to a neuronal differentiation medium composed of DMEM/F12 supplemented with N2, non-essential amino acids, and GlutaMAX™ (#17502048, #11330032, #35050061, #11140050, Thermo Fisher Scientific), together with human brain-derived neurotrophic factor (BDNF; 10 μg/L; #450–02-10 µg, PeproTech, Thermo Fisher Scientific Group), human neurotrophin-3 (NT-3; 10 μg/L; #450–03-10 µg, PeproTech, Thermo Fisher Scientific Group), and doxycycline. Puromycin (2 μg/mL; #P8833, Sigma-Aldrich) was added to the differentiation medium to select for successfully transduced, antibiotic-resistant cells. The following day (day 4), the differentiation medium was renewed and supplemented with mouse glial cells and cytosine β-D-arabinofuranoside (Ara-C; 2 g/L; #251010, Sigma-Aldrich) to limit astrocytic proliferation. Starting from day 7 of differentiation, neuronal cultures were treated with vehicle or hAMSC-EVs. EVs were added directly to the neuronal differentiation medium and maintained for a total duration of three consecutive weeks. Each well was treated with a defined number of EVs ranging from 6 × 10^6^ to 6 × 10⁷ particles, adjusted according to the number of plated cells to maintain a consistent EV-to-cell ratio (200:1). During this treatment period, EV supplementation was renewed at each medium change to ensure sustained exposure. Neurons were thus continuously exposed to vehicle, hAMSC-EV or hAMSC-EV_R_ until the end of the experimental protocol.

### Morphological analyses

Neuronal morphological development was longitudinally assessed using the Incucyte® SX5 Live-Cell Analysis System (Sartorius AG, Göttingen, Germany) equipped with the NeuroTrack (NT) analysis module. Neuronal cultures derived from sAD-hiPSCs and HS-hiPSCs were plated at a density of 80,000 cells/cm^2^ in 24-well plates and monitored up to 30 days following the initiation of differentiation (DIV30). Automated image acquisition was performed every 4 h using a 10 ×  objective, collecting four fields per well. Quantitative analyses included the evaluation of cell body cluster area (expressed as the total cluster area normalized to image area) and the total neurite length (sum of neurite lengths normalized to image area). NeuroTrack fluorescence analysis parameters were set as follows: acquisition channel (green), exposure time (200 ms), adaptive cell-body segmentation, threshold adjustment (10,000 GCU), hole filling (0 μm^2^), minimum cell width (7 μm), neurite coarse sensitivity (8), fine sensitivity (0.6), and neurite width (1 μm).

### hiPSCs characterization

The pluripotency of hiPSCs was assessed by immunocytochemical analysis using the StemLight™ Pluripotency Antibody Kit (#9656, Cell Signaling Technology). Cells were dissociated with StemPro™ Accutase™ Cell Dissociation Reagent (#A1110501, ThermoFisher Scientific), and 1 × 10^5^ cells were plated onto two-well glass chamber slides. After attachment, cells were fixed with 10% formalin solution (pH adjusted to 7.4 with NaOH) for 10 min at RT, followed by permeabilization with 0.3% Triton X-100 (Sigma-Aldrich) in PBS for 15 min. Non-specific antibody binding was blocked by incubating the samples for 20 min at RT in PBS containing 0.3% bovine serum albumin (#A2153, Sigma-Aldrich). Cells were then incubated overnight at 4 °C with primary antibodies diluted in the blocking solution. The following primary antibodies were used (all at 1:200 dilution): mouse anti-Oct4A (C30A3, #2840, Cell Signaling Technology), rabbit anti-SOX2 (D6D9, #3579, Cell Signaling Technology), rabbit anti-Nanog (D73G4 XP®, #5232, Cell Signaling Technology), mouse anti-SSEA4 (MC813, #4755, Cell Signaling Technology), and mouse anti-TRA-1–60(S) (#4746, Cell Signaling Technology). After primary antibody incubation, cells were washed in PBS and incubated for 90 min at RT with appropriate fluorophore-conjugated secondary antibodies diluted in blocking solution: Alexa Fluor 546 donkey anti-mouse, Alexa Fluor 546 donkey anti-rabbit, and Alexa Fluor 488 donkey anti-rabbit (all 1:1000; #A10036, #A10040, #A-21206, ThermoFisher Scientific). Nuclear counterstaining was performed by incubating the samples with DAPI (0.5 mg/mL, #D1306, ThermoFisher Scientific) in PBS for 10 min at RT. Coverslips were mounted using ProLong™ Gold antifade reagent (#36982, ThermoFisher Scientific), and samples were examined by confocal laser-scanning microscopy. Confocal images were acquired using a Nikon Ti Eclipse system at 20 × magnification with a resolution of 1024  ×  1024 pixels.

### Bioinformatics analysis

Functional enrichment analysis of the selected miRNAs was performed using the multiMiR and clusterProfiler R packages. For the human hAMSC-EV cargo, validated and predicted targets were retrieved for the human miRNAs; for the analyses referring to the murine model, the orthologs of the miRNAs enriched in the hAMSC-EV secretome were identified in miRBase on the basis of mature-sequence and seed identity, and their murine targets were retrieved with the same pipeline, so that the enrichment analysis was performed on mouse gene sets. Redundancy among enriched Gene Ontology (GO) terms was minimized using the rrvgo package. Bar plots were generated using the ggplot2 package. For the panel of AD-related genes, miRNA-target interactions were retrieved with multiMRI from both the validated (miRecord, miRTarBase, TarBase) and the predicted (DIANA-microT, EIMMo, MicroCosm, miRanda, miRDB, PicTar, POITA, TargetScan) sections of the database. Enriched terms were considered significant at an adjusted *P*<0.05 (Benjamini-Hochberg), and in all bar plots the bar length represent the number of predicted target genes associated with the term of pathway, not the enrichmenet significance.

### Statistical analysis

Sample sizes (*n* = 12 for behavioral tests; *n* = 4 for immunofluorescence experiments; *n* = 6 for multiplex and ELISA assays, *n* = 3–8 for western blotting) were chosen with adequate power (0.8) according to the results of prior pilot datasets or studies, including our own, that used similar methods or paradigms. Sample estimation and statistical analyses were performed using SigmaPlot 14 software. The choice of statistical test was based on the number of groups being compared and the experimental design. For comparisons between two independent groups, an unpaired two-tailed Student’s *t*-test was used. For comparisons of more than two groups, one-way analysis of variance (ANOVA) followed by appropriate post-hoc multiple-comparison tests was performed. When experiments involved two independent variables, two-way ANOVA followed by post-hoc multiple-comparison testing was used. The statistical tests employed in the experiments are mentioned in the corresponding figure legend. The level of significance was set at *P* < 0.05. Data are shown as mean ± SEM.

## Results

### hAMSC-EVs ameliorate AD neuropathology in female 3 × Tg-AD mice

AD represents the most common cause of dementia [28]. The prevalence of AD is greater in women than in men; accordingly, AD experimental models show sex-specific differences in terms of cognitive impairment, with female mice showing an earlier and more severe AD phenotype [29–31]. To evaluate the effects of hAMSC-EVs on the onset and progression of AD-related cognitive deficits, we isolated and characterized hAMSC-EVs. hAMSC-EVs showed a size range around 50–200 nm (with a peak at 105 nm; Fig. 1a), the EV-specific markers Alix, HSP70, CD81, CD9, and CD63 (Fig. 1b), and a typical cup shape (Fig. 1c). No signal was detected for cellular contamination marker GM130 (Fig. 1b).

**Fig. 2:**
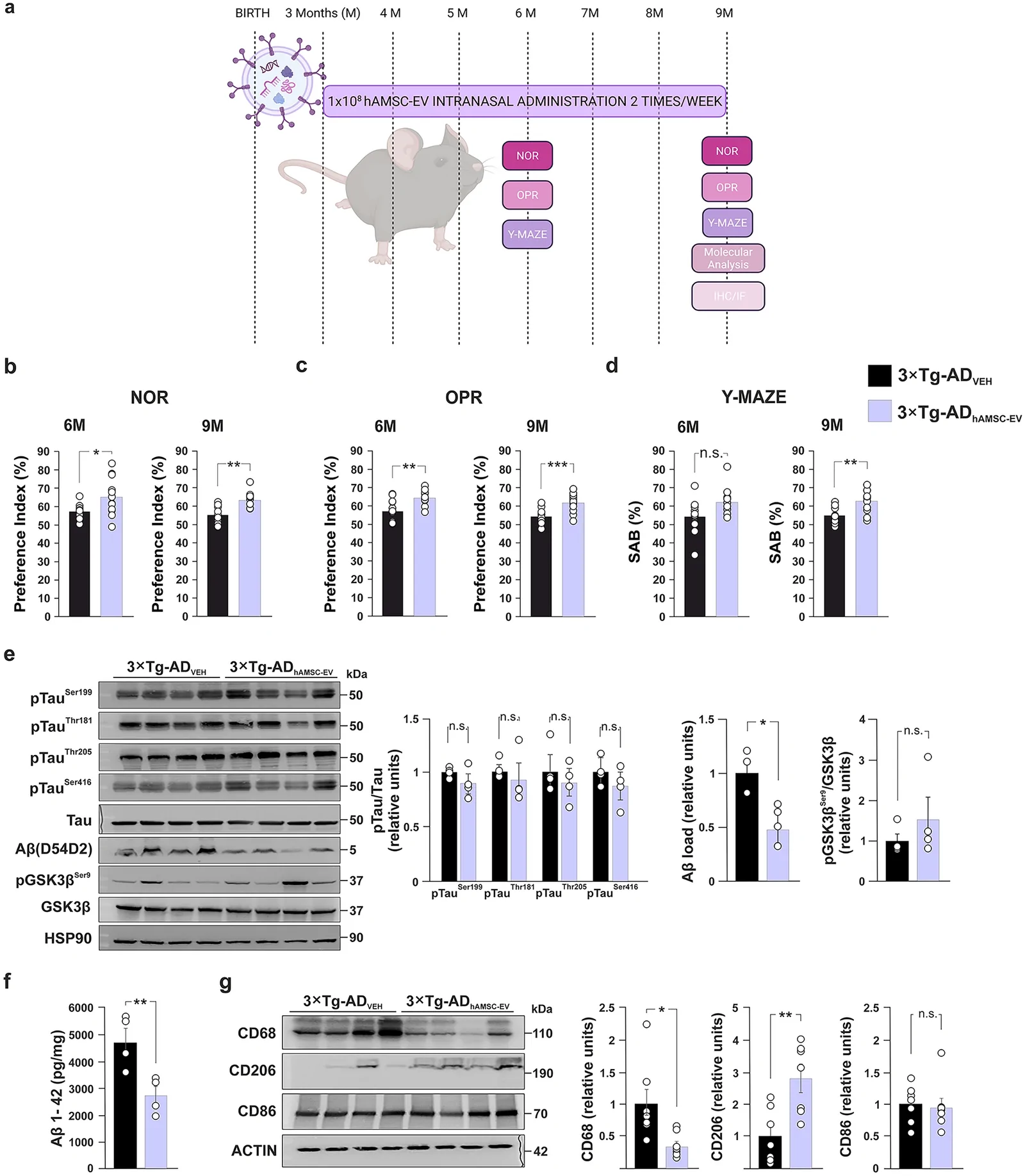
Intranasal hAMSC-EV treatment counteracts AD phenotype in 3 × Tg-AD female mice. **a** Experimental scheme of treatment of 3 × Tg-AD female mice and timeline of behavioral and molecular analyses. **b-d** Bar graphs showing preference indices in NOR test (**b**) and OPR test (**c**), and spontaneous alternation behavior (SAB) (**d**) in the Y-maze test for 6- and 9-month-old female 3×Tg-AD mice treated with vehicle solution (3×Tg-AD_VEH_) or hAMSC-EVs (3 × Tg-AD_hAMSC-EV_). *n* = 12 mice per group; unpaired Student’s *t-*test. **e** Representative immunoblots (left) and quantification (right) of levels of Aβ peptides, tau phosphorylation on different serine and threonine residues, and GSK3β phosphorylation in the hippocampus of 3 × Tg-AD_VEH_ and 3 × Tg-AD_hAMSC-EV_ mice. *n* = 4 mice per group; unpaired Student’s *t-*test. **f** Levels of Aβ_1-42_ peptide in the hippocampus of 3 × Tg-AD_VEH_ and 3 × Tg-AD_hAMSC-EV_ mice, analyzed by ELISA. The assay was performed in triplicate (*n* = 4 mice per group; unpaired Student’s *t-*test). **g** Representative immunoblots (left) and quantification (right) of the levels of CD68, CD206, and CD86 in the hippocampus of 3 × Tg-AD_VEH_ and 3 × Tg-AD_hAMSC-EV_ mice. *n* = 4 mice per group; unpaired Student’s *t-*test. Data are expressed as mean ± SEM. *P < 0.05; **P < 0.01; ***P < 0.001; n.s. not significant. NOR, Novel Object Recognition; OPR, Object Place Recognition; IHC, Immunohistochemistry; IF, Immunofluorescence

First, we checked whether intranasal administration allowed hAMSC-EVs to reach the hippocampus, as we had previously demonstrated for EVs derived from mouse neural stem cells [22]. Fluorescent dye-labeled hAMSC-EVs were found in all regions of the hippocampus, co-localized with both MAP2^+^ (i.e., neurons) and Iba1^+^ (i.e., microglia) cells (Fig. 1 d and e). The 3 × Tg-AD mouse model shows age-dependent AD-related behavioral and neuropathological alterations [32, 33], with female 3 × Tg-AD mice already exhibiting memory deficits at 6 months of age [25]. Therefore, we started treating female 3 × Tg-AD mice at 3 months of age, i.e., before the AD phenotype was manifested. Intranasal administration of either hAMSC-EVs or vehicle solution (PBS) was repeated twice a week, until 9 months of age, and cognitive functions were assessed by NOR, OPR and Y maze tests after 3 and 6 months of treatment, corresponding to 6- and 9-month age, respectively (Fig. 2a).

Administration of hAMSC-EVs significantly ameliorated cognitive performance, as assessed by the preference index (PI) in the NOR and OPR tests, after both 3 and 6 months of treatment (3 months, NOR: 65.19% vs. 57.29%, *P* = 0.0207; OPR: 64.44% vs. 57, 19%, *P* = 0.0025; 6 months, NOR: 63.29% vs. 55.23%, *P* = 0.0002; OPR: 61.44% vs. 54.06%, *P* = 0.0006; Fig. 2b and c). Moreover, spontaneous alternation behavior in the Y-maze paradigm was significantly increased in 3 × Tg-AD mice after 6-month treatment with hAMSC-EVs (62.5%±1.9% for 3 × Tg-AD_hAMSC-EV_ vs. 54.8%±1.3% for 3 × Tg-AD_VEH_, *P* = 0.0017; Fig. 2d). Aβ deposition and tau phosphorylation represent fundamental hallmarks of AD phenotype in both human and mouse brains [34]. Here, immunoblotting analysis revealed reduced levels of Aβ in 9-month-old female 3 × Tg-AD mice after intranasal administration of hAMSC-EVs (− 53%, *P* = 0.021; Fig. 2e). Consistently, ELISA assay showed decreased amounts of synaptotoxic Aβ_1-42_ peptides in the hippocampus of 3 × Tg-AD_hAMSC-EV_ mice compared to 3 × Tg-AD_VEH_ (2743.7±341.2 pg/mg vs. 4712.5±483.7 pg/mg, *P* = 0.0085; Fig. 2f). We analyzed the expression of both pro-amyloidogenic secretase BACE1 and main enzymes involved in Aβ clearance in the hippocampus of 3 × Tg-AD_hAMSC-EV_ mice, but no changes were detected compared to 3 × Tg-AD_VEH_ animals (Fig. S1a). However, immunoblot analysis of microglia markers revealed lower expression of proinflammatory CD68 and higher levels of mannose receptor CD206 associated with anti-inflammatory state in 3 × Tg-AD_hAMSC-EV_ hippocampi, suggesting a potential shift toward the M2 state of microglia upon hAMSC-EV administration (CD68 − 64.8%, *P* = 0.0215; CD206 +180.9%, *P* = 0.0047; Fig. 2g). No significant difference was evident for the expression of CD86 (Fig. 2g). We also investigated the phosphorylation of tau at different activity-related serine (Ser) and threonine (Thr) residues, and the phosphorylation of GSK3β kinase, but no significant changes were detected in the hippocampus of 3 × Tg-AD_hAMSC-EV_ animals (Fig. 2e).

Finally, we tested the therapeutic potential of hAMSC-EVs through intranasal administration in 9-month-old 3 × Tg-AD mice (when AD phenotype has already manifested) for one month and evaluated the cognitive functions at the end of treatment. The 10-month-old AD mice showed an improvement of memory deficits after hAMSC-EV administration in NOR test but not in OPR test (Fig. S1b).

Collectively, our findings suggest that intranasal administration of hAMSC-EVs ameliorates cognitive deficits and prevents hippocampal Aβ deposition in female 3 × Tg-AD mice, potentially modifying the activation state of microglia.

### hAMSC-EV treatment reduces glial reactivity and alters microglial morphology

**Fig. 3:**
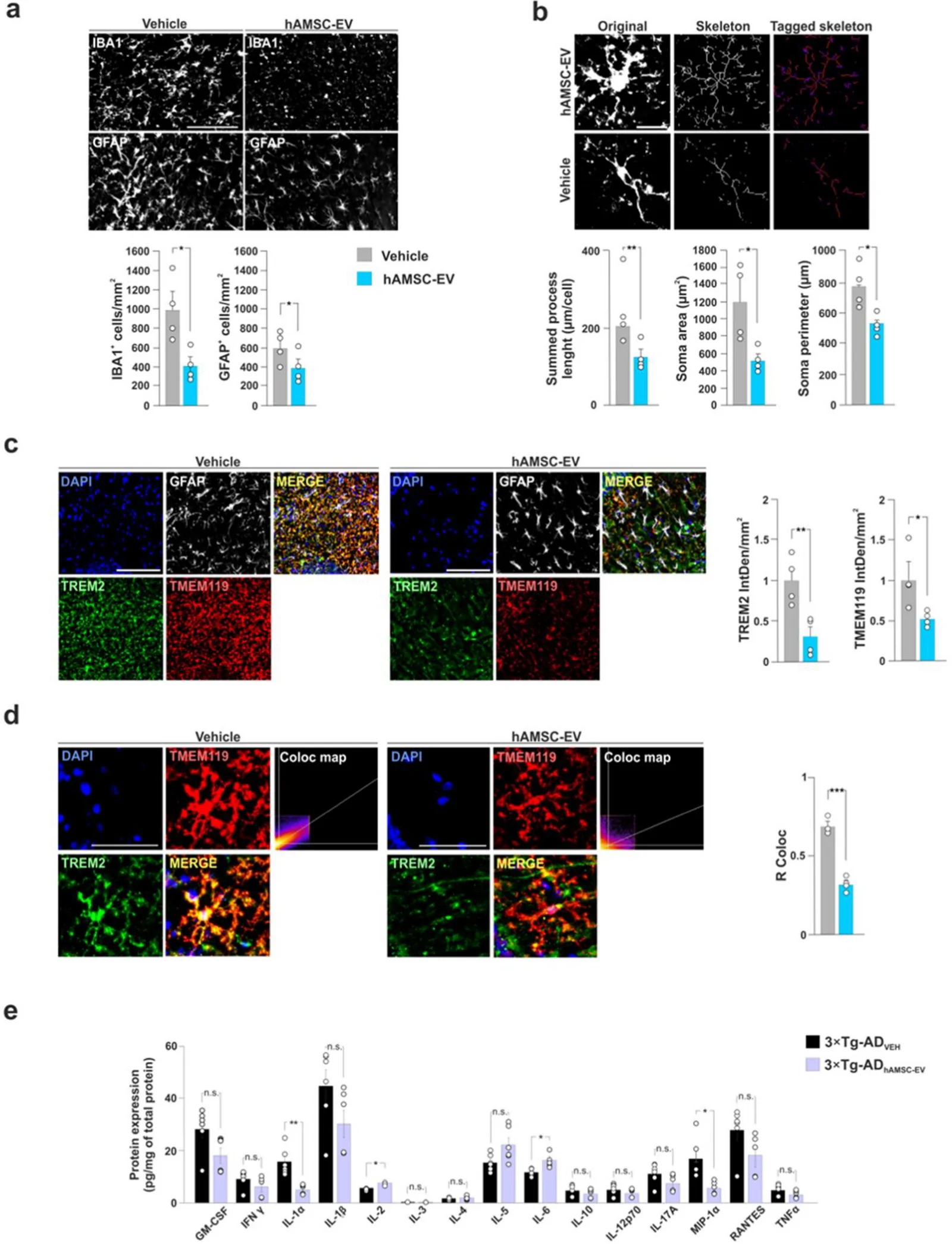
hAMSC-EV treatment attenuates astrocyte reactivity and induces microglia structural remodeling in 3 × Tg-AD female mice. **a** Representative images and quantification of Iba1⁺ microglia and GFAP⁺ astrocytes in brain sections from 3 × Tg-AD_VEH_ and 3 × Tg-AD_hAMSC-EV_ mice (*n* = 4 mice per group; unpaired Student’s *t-*test. Cell densities were normalized to tissue area (cells/mm^2^). Scale bar: 100 μm. **b** Representative high-magnification images of Iba1⁺ microglia processed for skeleton-based morphometric analysis, and quantitative analysis of total process length, soma area, and perimeter in brain sections from 3 × Tg-AD_VEH_ and 3 × Tg-AD_hAMSC-EV_ mice. Images were binarized and skeletonized using ImageJ. Branches (orange), endpoints (blue), and junctions (purple) are color-coded to illustrate structural features. *n* = 4 mice per group; unpaired Student’s *t-*test. Scale bars: 10 μm. **c** Representative immunofluorescence images showing TMEM119 (− 51.17% ± 5.04%, *P* = 0.0355) and TREM2 (− 69.33% ± 12.51%, *P* = 0.0048) staining in brain sections from 3 × Tg-AD_VEH_ and 3 × Tg-AD_hAMSC-EV_ mice (*n*=4 mice per group; unpaired Student’s *t-*test). Scale bars: 100 μm. **d** Higher magnification of selected regions from panel **c** highlighting TMEM119-TREM2 signal colocalization in 3 × Tg-AD_VEH_ and 3 × Tg-AD_hAMSC-EV_ mice (*n*=4 mice per group; unpaired Student’s *t-*test; Scale bars: 25 μm). Quantitative colocalization analysis using the Coloc plugin in ImageJ revealed a significant decrease in the colocalization coefficient (*R*) in the hAMSC-EV group compared to vehicle controls. **e** Levels of GM-CSF, IFNγ, IL-1α (− 68.7% ± 1.9% *P* = 0.00654), IL-1β, IL-2 (+ 35.1% ± 6.3%, *P* = 0.02405), IL-3, IL-4, IL-5, IL-6 (+ 40.1% ± 5.8%, *P* = 0.01889), IL-10, IL-12p70, IL-17, MIP-1α (− 66.8% ± 2.8%, *P* = 0.02405), RANTES, and TNFα in hippocampal tissues of 3 × Tg-AD_VEH_ and 3 × Tg-AD_hAMSC-EV_ mice. *n* = 6 female mice per group; multiple Student’s *t* tests followed by Benjamini–Hochberg false discovery rate procedure. Multiplex analyses were performed in triplicate. Data are expressed as mean ± SEM. *P < 0.05; **P < 0.01; ***P < 0.001; n.s. not significant

Multiple experimental, neuropathological and epidemiological evidence indicates a critical role for neuroinflammation in AD [14]. Moreover, intranasal delivery of hAMSC conditioned medium (CM-hAMSC) has been reported to exert anti-inflammatory and neuroprotective effects in experimental models of neurodegenerative disorders [35, 36]. To determine the impact of hAMSC-EVs on glial responses in our AD mouse model, quantitative immunofluorescence analyses of Iba1⁺ microglia and GFAP⁺ astrocytes were performed in AD vulnerable brain regions. Upon hAMSC-EV treatment, significant reductions in the numbers of both Iba1⁺ and GFAP⁺ cells were observed in the hippocampus and entorhinal cortex compared to the vehicle-treated controls (Iba1^+^ cells: 406.61±92.62 vs. 988.67±191.08, *P* = 0.0193; GFAP^+^ cells: 404.14±15.37 vs. 611.18±94.08, *P* = 0.0458; Fig. 3a). We also analyzed the impact of hAMSC-EV treatment on microglia morphology. High-resolution imaging revealed that the Iba1⁺ microglia in vehicle-treated mice exhibited highly ramified structures, characterized by elaborated and branched processes and enlarged soma. Conversely, microglia from the hAMSC-EV-treated animals displayed less complex morphologies, including retraction of processes and reduced branching. Upon hAMSC-EV administration, the morphotype ranged from ameboid, with rounded soma and minimal process extension, to moderately ramified profiles with limited arborization (Fig. 3a).

To quantify these morphological changes, a skeleton-based morphometric analysis was performed on binarized and skeletonized confocal image stacks. Microglia from the hAMSC-EV-treated animals exhibited a significant decrease in the total process length compared to controls, indicating reduced branching complexity (127.26 ± 18.39 μm/cell vs. 204.54±12.72 μm/cell, *P* = 0.0071; Fig. 3b). Moreover, decreases in soma area and perimeter were observed, reflecting attenuation of microglial hypertrophy (soma area: 522.95 ± 73.05 μm^2^ vs. 1202.59 ± 258.62 μm^2^, *P* = 0.0265; soma perimeter: 525.87 ± 34.93 μm vs. 775.37 ± 77.30 μm, *P* = 0.0144; Fig. 3b).

To determine whether hAMSC-EV administration influenced the molecular profile of microglia, immunofluorescence analysis of TMEM119 and TREM2 was conducted on brain sections from 3 × Tg-AD mice. Quantitative assessment revealed a significant reduction in the fluorescence intensity of both markers in 3 × Tg-AD_hAMSC-EV_ compared to controls (Fig. 3c). This reduction was consistently observed in the hippocampus and the entorhinal cortex of treated mice.

To further assess potential changes in microglial identity, a colocalization analysis of TMEM119 and TREM2 signals was performed using the Coloc 2 plugin in ImageJ. A marked decrease in the co-localization of the two markers was detected in the 3 × Tg-AD_hAMSC-EV_ animals, as indicated by significantly lower Pearson’s correlation coefficients and a reduced proportion of colocalized pixels (R colocalization coefficient: 0.32±0.03 vs 0.68±0.03, *P* = 0.0001; Fig. 3d). This decrease in colocalization was accompanied by qualitative differences in marker distribution patterns, with the treated animals exhibiting reduced spatial clustering and more dispersed labeling profiles.

Finally, we investigated whether treatment with hAMSC-EVs modulated the inflammatory response in the brains of 3 × Tg-AD mice. Levels of both proinflammatory and anti-inflammatory cytokines in the hippocampal tissue of 3 × Tg-AD mice were analyzed_._ We found that the hAMSC-EV treatment significantly altered the local cytokine profile, leading to a reduced amount of some proinflammatory cytokines and increased levels of the main anti-inflammatory cytokines in 3 × Tg-AD_hAMSC-EV_ (Fig. 3e). No changes were detected in the levels of other cytokines (Fig. 3e).

Collectively, these data indicate that hAMSC-EV administration reduces hippocampal neuroinflammation in female 3 × Tg-AD mice by inducing microglia structural remodeling and attenuating the activation of both astrocytes and microglia.

### miRNA profiling reveals dual neuroprotective and immunomodulatory effects of hAMSC-EVs

To better understand the mechanisms underlying the therapeutic effects of hAMSC-EVs, we reanalyzed the miRNA profile characterized in our previous studies [18, 19], to investigate whether its molecular composition could help explain the neuroprotective and immunomodulatory effects observed in our in vivo models.

**Fig. 4:**
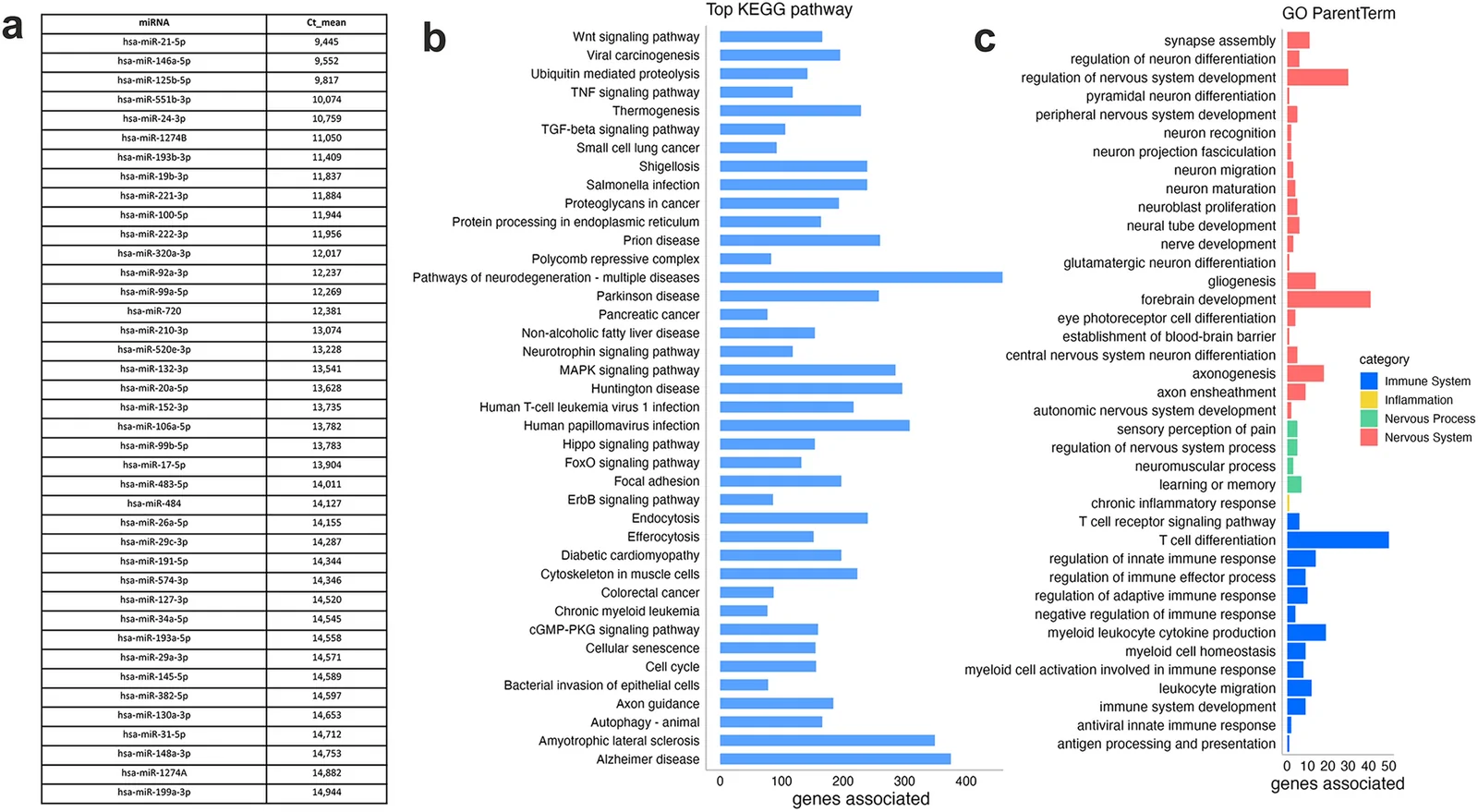
miRNA profiling and functional enrichment analysis of the top-ranking miRNAs in the hAMSC-EVs. **a** Top 40 most abundant human miRNAs identified in the hAMSC-EVs, ranked by the mean expression levels across replicates. **b** KEGG pathway enrichment analysis of predicted miRNA targets, highlighting significant associations with neurodegenerative disorders (Alzheimer’s disease, Parkinson’s disease, Huntington’s disease) and relevant intracellular signaling pathways. **c** GO Biological Process enrichment analysis for immune system (blue), inflammation-related (yellow) pathways, and nervous system-related categories, including nervous system development (red) and neurobiological functions (green). Results indicate strong involvement of miRNA targets in leukocyte migration and inflammatory response pathways, as well as notable enrichment in axon guidance, synapse assembly, and cognitive processes such as learning and memory

Exploratory analysis of the 40 most abundant miRNAs, based on raw mean Ct values, revealed a profile strongly enriched in conserved regulatory molecules, including the well-known immunomodulatory and neuroprotective miRNAs hsa-miR-21-5p, hsa-miR-146a-5p, hsa-miR-125b-5p and hsa-miR-100-5p (Fig. 4a).

Kyoto Encyclopedia of Genes and Genomes (KEGG) pathway enrichment analysis showed a clear convergence on processes associated with neurodegeneration. AD, pathways of neurodegeneration, and ubiquitin-mediated proteolysis emerged as top hits. These findings suggest that the miRNA cargo may contribute to limiting proteinopathy-associated toxicity (Fig. 4b).

Further GO biological process (BP) analysis was performed for four specific categories of interest, “immune system”, “inflammation”, “nervous system” and “nervous process”. Results revealed two main biological axes: one associated with neuronal repair and plasticity (including axonogenesis, synapse formation, and memory-related processes) and another centered on immune modulation, particularly the regulation of inflammatory pathways and T-cell differentiation (Fig. 4c).

To test whether these miRNAs are relevant to AD pathology, we focused on a panel of key genes involved in disease progression (BACE1, APP, MAPT, and NLRP3). Cross-referencing this panel with the multiMIR interaction data assigned a distinct subset of cargo miRNAs to each gene.

The strongest evidence supports the amyloidogenic arm. miR-17-5p, miR-20a-5p and miR-106a-5p target APP, and miR-29a-3p targets BACE1; all four interactions were validated by reporter assay with a concomitant decrease in endogenous protein. For MAPT, miR-483-5p is experimentally supported, whereas miR-34a-5p and miR-320a-3p are predicted by five and four independent algorithms, respectively. NLRP3 is targeted by miR-193b-3p and miR-100-5p, although these interactions rest on weak functional evidence only.

Taken together, distinct cargo components converge on the amyloidogenic, tau-related and inflammatory arms of AD pathology, in line with the immune-related biological processes shown in Fig. 4c.

Overall, these analyses suggest that hAMSC-EVs carry a functionally coherent miRNA cargo capable of acting on multiple fronts, thus supporting the idea that their therapeutic action is not mediated by a single mechanism, but rather by modulation of immune responses along with preservation of synaptic integrity.

### hAMSC-EV administration enhances the neuroplasticity-associated signaling in the hippocampus of 3 × Tg-AD mice

Administration of hAMSC-EVs changed the inflammatory cytokine profile in the hippocampus of 3 × Tg-AD mice. Aberrant neuroinflammation has been proposed to affect neuronal integrity and synaptic function, leading to the progression of neurodegeneration [11, 12]. To identify miRNAs potentially counteracting the neuroinflammation-mediated detrimental effects, we reanalyzed previously published transcriptomic data comparing the EV miRNA cargo from hAMSCs, bone marrow-derived MSCs, and adipose tissue-derived MSCs [18]. In the list of miRNAs significantly upregulated in the hAMSC-EVs compared to the other EVs, there are three miRNAs known to be involved in multiple biological processes associated with neuroinflammation and neurological disease, including miR-146a-5p, miR-410-3p, and miR-126-3p.

To assess the functional relevance of these miRNAs in vivo, we first identified their murine counterparts. According to miRBase, hsa-miR-146a-5p, hsa-miR-410-3p and hsa-miR-126-3p each have a single murine ortholog (mmu-miR-146a-5p, mmu-miR-410-3p and mmu-miR-126a-3p) whose mature sequences, and therefore whose seed regions, are identical to that of the corresponding human miRNAs. Consistently, the human and murine forms share 53%–75% of their conserved predicted target genes, indicating that a human miRNA delivered by hAMSC-EVs and its murine ortholog are expected to engage the same transcripts in the recipient mouse tissue. This sequence correspondence is what allows the biological effects of the human EV cargo to be interpreted in the murine model.

**Fig. 5:**
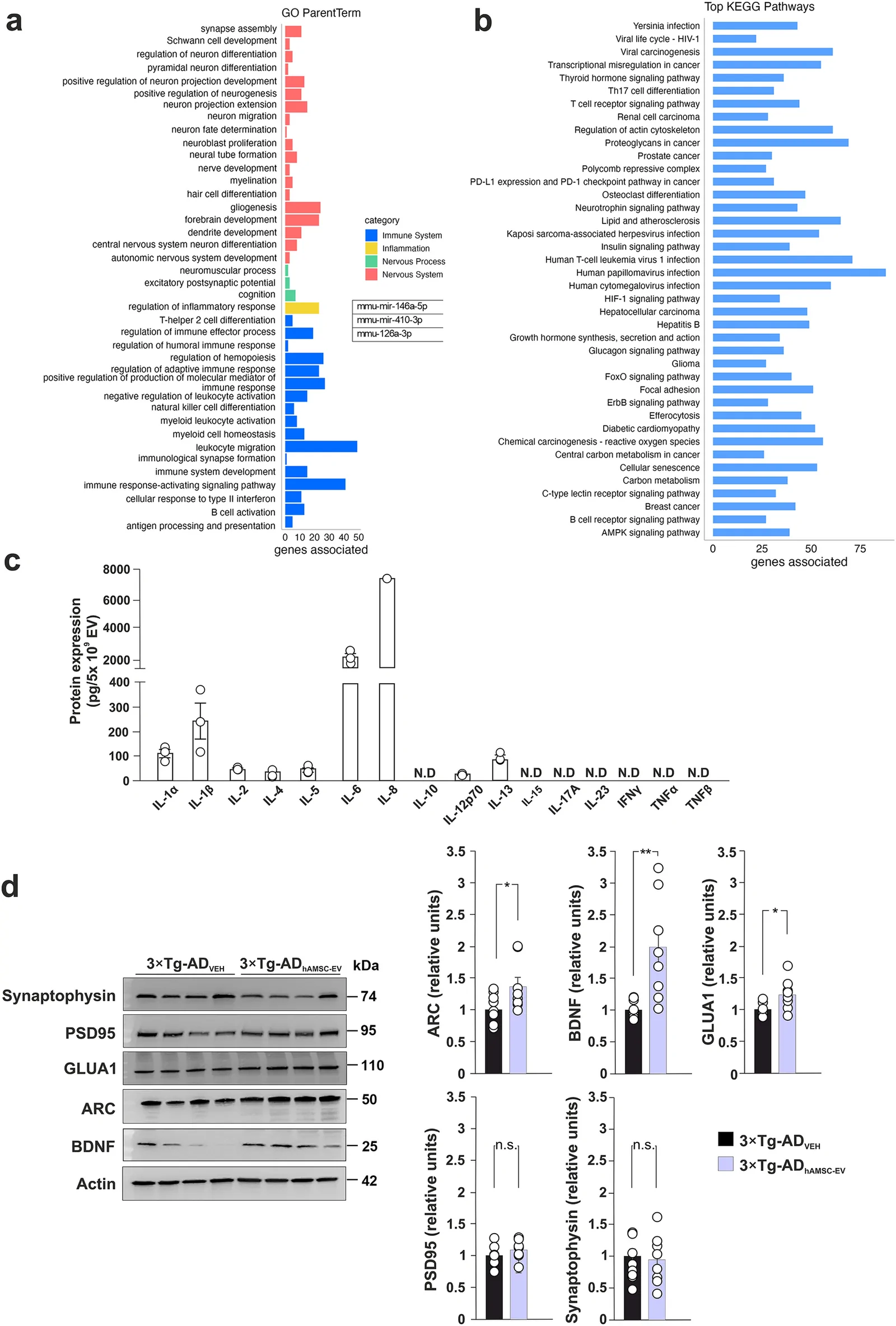
hAMSC-EV cargoes modulate neuroplasticity-related enzymes in the hippocampus of 3 × Tg-AD mice. **a** GO biological process analysis of mmu-miR-146a-5p, mmu-miR-410-3p, mmu-miR-126-3p, murine counterparts of miR-146a-5p, miR-410-3p, and miR-126-3p that are significantly upregulated in the EV secretome of human amniotic mesenchymal stromal cells (hAMSCs) compared to bone marrow- and adipose-derived MSCs. The enrichment analysis was focused on pathways linked to immune system, inflammation, nervous system and process. Reduction to parent terms was used to reduce redundancy among enriched terms. **b** KEGG pathway enrichment highlighted signaling pathways implicated in neurodegeneration, axon guidance, immune regulation, cellular metabolism, and inflammatory responses, including neurotrophin, FoxO, T and B cell receptor, and AMPK signaling pathways. Bars represent the number of miRNA-targeted genes associated with each pathway. **c** Concentrations of IL-1α, IL-1β, IL-2, IL-4, IL-5, IL-6, IL-8, IL-10, IL-12p70, IL-13, IL-15, IL-17A, IL-23, IFNγ, TNFα, and TNF-β (*n *= 3 independent EV preparations). Each hAMSC-EV sample was read in triplicate. 5×10^9^ EVs were loaded in each well. Data are expressed as mean ± SEM. **d** Representative immunoblots and quantification of the expression levels of plasticity-related proteins in the hippocampus of 3 × Tg-AD_VEH_ and 3 × Tg-AD_hAMSC-EV_ mice (*n* = 8 mice per group; ARC +37.3% ± 14.1%, *P* = 0.0384; GluA1 +24.3% ± 8.5%, *P* = 0.0205; BDNF + 98.7% ± 28.7%, *P* = 0.0044; unpaired Student’s *t*-test). Data are expressed as mean ± SEM. *P < 0.05; **P < 0.01; ***P < 0.001; n.s. not significant; N.D. not detectable

The predicted murine target genes of the three orthologs were therefore retrieved and interrogated through two complementary enrichment approaches. GO analysis, restricted to biological process categories linked to the immune system, inflammation and the nervous system, revealed enrichment in terms related to nervous system development (e.g., regulation of nervous system development, neuron maturation, gliogenesis), cognitive processes (learning or memory) and immune function (e.g., T cell differentiation, leukocyte migration, innate and adaptive immune regulation). Notably, inflammatory pathways such as positive regulation of inflammatory response were also represented, indicating the capacity of these miRNAs to orchestrate both neurogenic and immunomodulatory responses (Fig. 5a). KEGG pathway analysis further showed significant enrichment in signaling pathways relevant to neurodegeneration and immune regulation (Fig. 5b), including neurotrophin, glioma, FoxO and ErbB signaling, all linked to neuronal survival and plasticity, as well as B and T cell receptor signaling and efferocytosis, reflecting immunoregulatory functions. Additional pathways associated with stress responses (HIF-1 and AMPK signaling) and metabolic regulation were also enriched. It should be noted that both enrichment analyses were performed on genome-wide miRNA–target prediction databases and were therefore not restricted, by design, to transcripts expressed in the hippocampus: they define the biological processes that the hAMSC-EV miRNA cargo is intrinsically able to engage, irrespective of the recipient tissue.

Moreover, cytokine profile analysis within the hAMSC-EV cargo revealed measurable amounts of IL-1α, IL-1β, IL-2, IL-4, IL-5, IL-6, IL-8, IL-12p70, and IL-13 (cytokine levels/5 × 10^9^ EVs: 111.9 ± 20.9 pg for IL-1α; 243.9 ± 89.9 pg for IL-1β; 46.1 ± 3.7 pg for IL-2; 22.8 ± 10.8 pg for IL-4; 29.7 ± 11.1 pg for IL-5; 2178.6 ± 302.7 pg for IL-6;  > 3760 pg for IL-8; 18.1 ± 2.9 pg for IL-12p70; 95.4 ± 12.8 pg for IL-13; Fig. 5c). Other cytokines, such as IL-10, IL-15, IL-17A, IL-23, IFNγ, TNFα, and TNFβ, could not be assessed because their levels were lower than the detection threshold of the methodology employed.

Finally, we wondered if the beneficial effects of hAMSC-EV treatment on memory were due to the increased levels of key proteins regulating neuronal integrity and synaptic function. Immunoblotting analysis showed that hAMSC-EVs increased the levels of proteins regulating neuroplasticity, such as ARC, GluA1 and BDNF (Fig. 5d). No changes were detected in the expression of proteins like PSD95 and synaptophysin (SYP). Collectively, our data indicate that hAMSC-EVs contain non-coding RNAs and immunomodulatory cytokines, potentially promoting the expression of pro-neuroplasticity enzymes and preventing the neuroinflammation-mediated neurodegeneration in the hippocampus of 3 × Tg-AD mice.

### hAMSC-EVs reduce microglial uptake of Aβ and attenuate their activation via RNA-based mechanisms

**Fig. 6:**
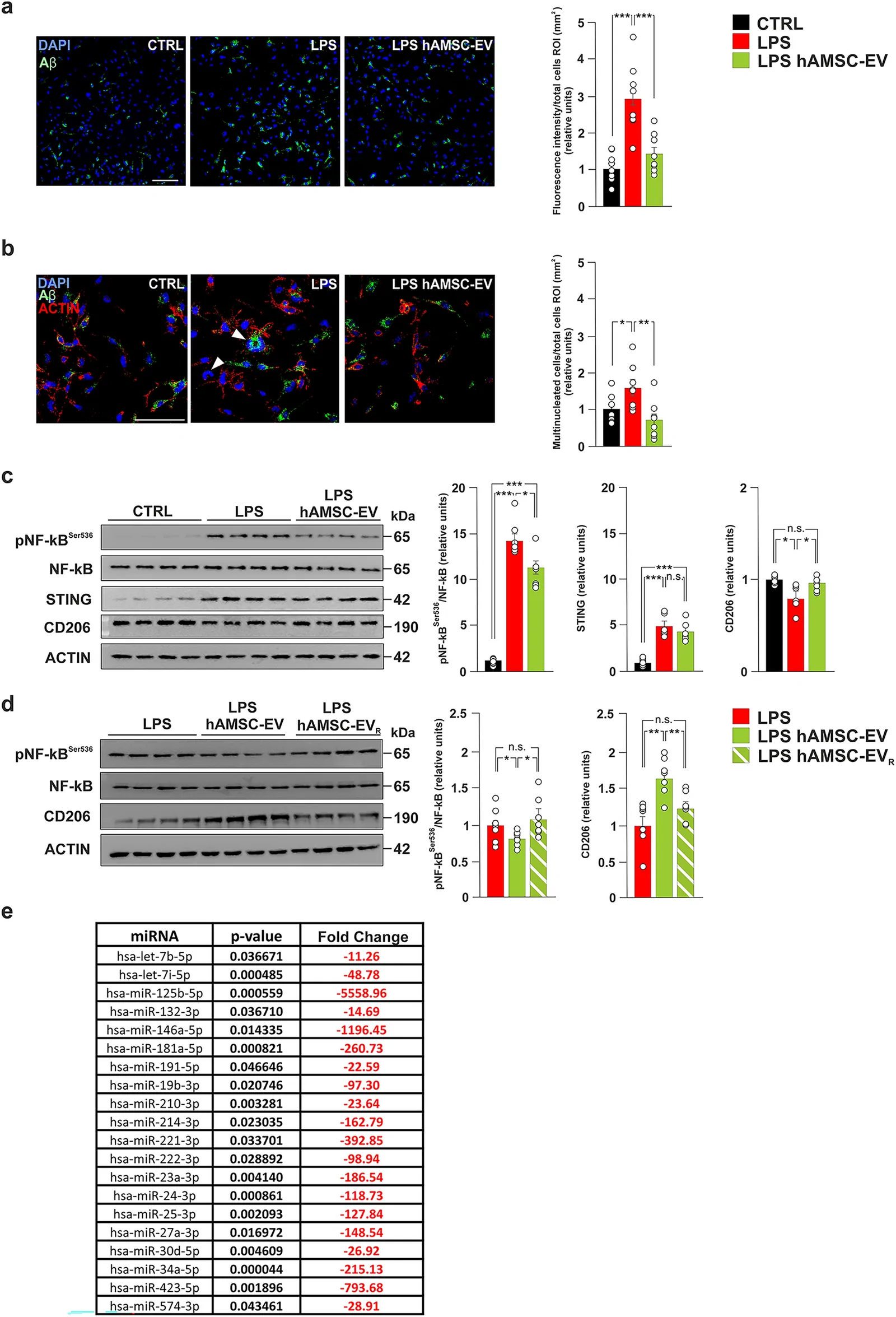
hAMSC-EVs reduce microglia activation via RNA-based mechanisms. **a** Immunostaining and quantification of Aβ uptake in vehicle-, LPS- and LPS hAMSC-EV-treated microglial cells (*n* = 8 ROI per experimental group; two-way ANOVA and Bonferroni post-hoc test). Experiments were performed in triplicate. **b** Immunostaining and quantification of the number of multinucleated microglial cells treated with vehicle, LPS and LPS hAMSC-EVs (*n* = 8 ROI per experimental group; two-way ANOVA and Bonferroni post-hoc test). Scale bar: 50 µm. **c** Immunoblots and quantification of the expression of phospho-NF-kB, STING, and CD206 proteins in vehicle-, LPS- and LPS hAMSC-EV-treated microglia (*n*=7 per experimental group; two-way ANOVA and Bonferroni post-hoc test). **d** Immunoblots and quantification of the expression of phospho-NF-kB and CD206 proteins in LPS-, LPS hAMSC-EV-, and LPS hAMSC-EV_R_-treated microglia (*n* = 6 per experimental group; pNF-kB: *F*_3.885_=4.96, *P* = 0.572 for LPS vs. LPS hAMSC-EV_R_; CD206: *F*_3.554_=9.59, *P* = 0.191 for LPS vs. LPS hAMSC-EV_R_; two-way ANOVA and Bonferroni post-hoc test). **e** *P*-values and fold changes of the most representative inflammation-related miRNAs depleted in hAMSC-EVs after treatment with digitonin and RNAse (*n* = 4 EV preparations). Data are expressed as mean ± SEM. *P < 0.05; **P < 0.01; ***P < 0.001; n.s. not significant

To understand the role of microglia modification in the beneficial effects of hAMSC-EV treatment, we set up an in vitro model of microglial activation induced by LPS and evaluated the uptake of fluorescently-labeled Aβ. Immunofluorescence analysis revealed that pre-treatment with hAMSC-EVs reduced the LPS-induced Aβ uptake (− 51.5%, *P* = 0.0005; Fig. 6a) and the number of reactive polynucleate cells (− 55.7%, *P* = 0.0053; Fig. 6b).

Immunoblot analysis showed that hAMSC-EVs decreased pNF-kB level (− 25.7%, *F*_4.102_=163.84, *P* = 0.0101) and increased the level of CD206 (+ 23.6%, *F*_4.102_=4.83, *P* = 0.0387) compared to the LPS treatment group (Fig. 6c). No significant effect of hAMSC-EVs was detected for STING (Fig. 6c). To investigate the role of the RNA cargoes in hAMSC-EVs in the modulation of microglial activation, we removed large part of RNAs from hAMSC-EV cargo by permeabilizing the vesicles with digitonin and treating them with RNAse (hAMSC-EV_R_). hAMSC-EV_R_ lost the capability to attenuate the LPS-dependent activation of microglia compared to naïve hAMSC-EVs (Fig. 6d).

Finally, we analyzed the cargoes of hAMSC-EV_R_ and confirmed a significant reduction of inflammation-related miRNAs such as miR-125b-5p, miR-146a-5p, miR-181a-5p, miR-221-3p, and miR-423-5p (Fig. 6e and Table S2).

Collectively, our data revealed that RNA-based mechanisms underlie the ability of hAMSC-EVs to dampen microglial activation.

### hAMSC-EVs counteract the morphological and molecular alterations of human AD neurons

**Fig. 7:**
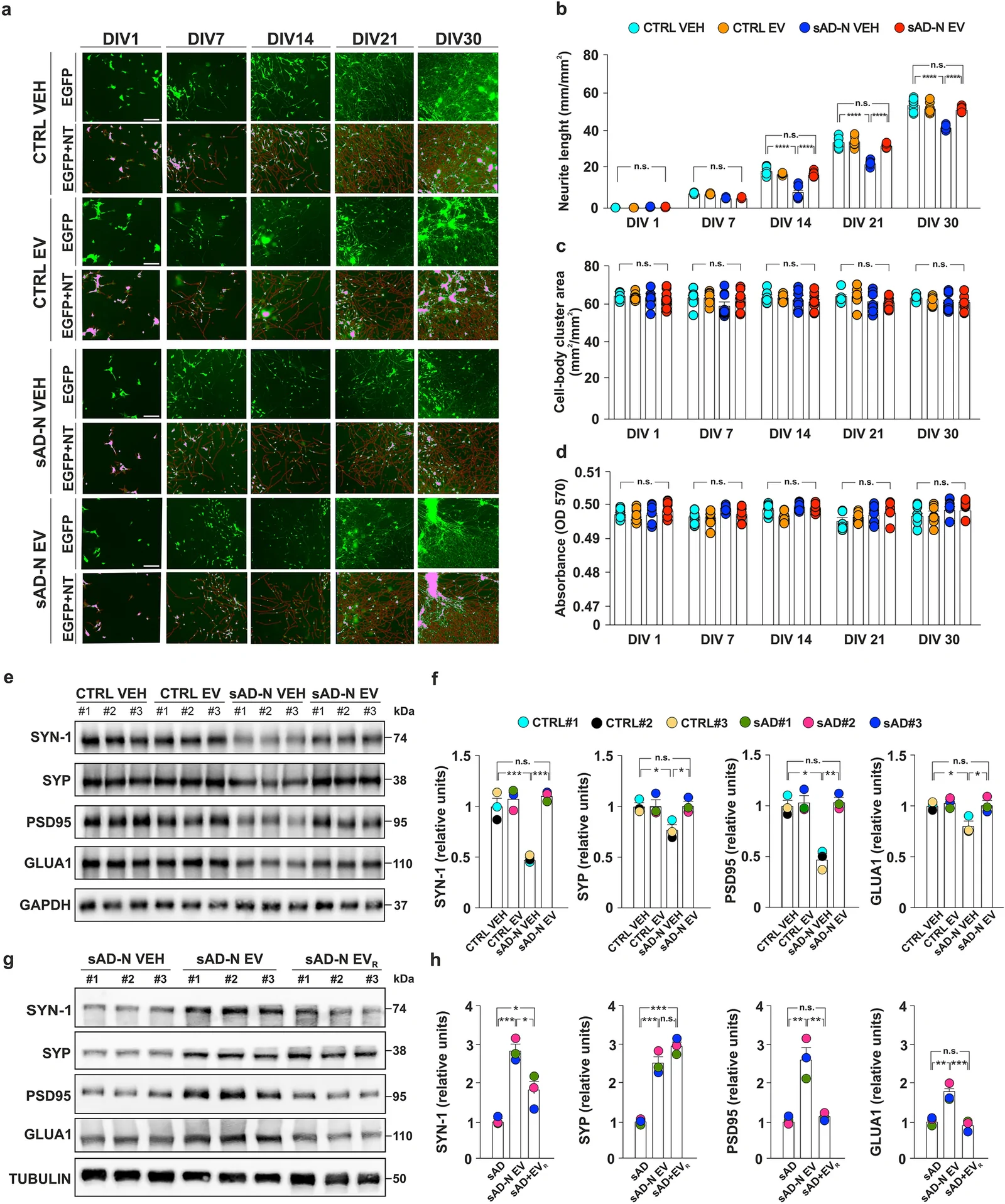
hAMSC-EVs prevent alterations of both neurite length and neuroplasticity-related proteins in sAD-N. **a** Representative images at DIV1, DIV7, DIV14, DIV21, and DIV30 of hiPSC-derived neurons obtained from healthy subjects (CTRL) and sAD patients (sAD) and treated with vehicle (VEH) or hAMSC-EV (EV). Fluorescence-based NeuroTrack analysis was performed via the IncuCyte® time-lapse system by exploiting EGFP expression in hNs. Cell body clusters are marked in pink, neurites in red. Scale bar, 50 μm. **b**, **c** Neurite length per neuron (**b**) and cell body cluster area (**c**) analyzed at DIV1, DIV7, DIV14, DIV21, and DIV30 in CTRL VEH, CTRL EV, sAD-N VEH, and sAD-N EV neurons (*n* = 3 subjects with *n* = 3 biological replicates per subject for any experimental group; two-way repeated measures ANOVA and Bonferroni post-hoc test). **d** Cell viability evaluated by MTT assay in CTRL VEH, CTRL EV, sAD-N VEH, and sAD-N EV neurons (*n*=3 subjects with *n*=3 biological replicates per subject for any experimental group; two-way repeated measures ANOVA and Bonferroni post-hoc test). Each data point in panels **b-d** represents a single neuronal cell culture preparation with each measurement performed in technical triplicate (3 replicates per group). **e**, **f** Representative immunoblots and quantification of the expression of presynaptic (SYN-1, SYP) and postsynaptic (PSD95, GLUA1) proteins at DIV30 in CTRL VEH, CTRL EV, sAD-N VEH, and sAD-N EV neurons (*n* = 3 subjects per experimental group; two-way ANOVA and Bonferroni post-hoc test). **g** Representative immunoblots and quantification of the expression of SYN-1, SYP, PSD95, and GLUA1 proteins at DIV30 in neurons treated with sAD-N VEH, sAD-N EV, or RNA-depleted sAD-N EV (sAD-N EV_R_) (*n* = 3 subjects per experimental group; one-way ANOVA and Brown-Forsythe post-hoc test). Data are presented as mean ± SEM. *P < 0.05; **P < 0.01; ***P < 0.001; n.s. not significant

We further evaluated the translational potential of hAMSC-EV treatment in human AD. Fibroblasts isolated from skin biopsies of sAD patients and age-matched healthy controls were reprogrammed into hiPSCs, which were differentiated into glutamatergic neurons. We previously characterized the sAD-derived neurons (sAD-N) and reported that they showed reduced neurite length starting at DIV14 of differentiation compared to control neurons derived from healthy subjects [24]. To obtain proof of concept about the therapeutic potential of hAMSC-EVs in human AD, vehicle or hAMSC-EVs were added to the culture media during hiPSC differentiation into glutamatergic neurons (Fig. 7a). Morphological analysis revealed a significant decrease in neurite length of sAD-N compared to control groups starting from DIV14 (*F*_1.81_=25.79, DIV14: −60.3% ± 8.9%, *P* = 2.89 × 10^–7^; DIV30: −21.8% ± 1.1%, *P* = 1.19 × 10^–6^; Fig. 7a and b). Notably, hAMSC-EV treatment prevented the alterations of neuronal morphology in sAD-N (*F*_1.81_=25.79, DIV14: +128.7% ± 2.6%, *P* = 3.68 × 10^–6^; DIV30: +22.09%±1.6%, *P* = 6.27 × 10^–11^; Fig. 7a and b). No significant changes in cell body clusters were detected in any experimental condition (Fig. 7c). Moreover, MTT assay did not reveal any change in cell viability from DIV1 to DIV30 upon hAMSC-EV administration (Fig. 7d).

Finally, to investigate the molecular changes in sAD-N upon hAMSC-EV treatment, we analyzed the expression of key neuroplasticity-related proteins at the end of differentiation protocol (i.e., DIV30). Immunoblot analysis revealed lower levels of pre- and post-synaptic proteins in sAD-N compared to controls, including synapsin-1 (SYN-1), SYP, PSD95, and GluA1 (SYN-1 −52.5%, SYP −23.1%, PSD95: −51.4%, GluA1: −19.7%; Fig. 7e and f). More importantly, hAMSC-EV treatment increased the levels of these proteins in sAD-N (SYN-1 +131.5%, *F*_4.75_= 57.51, *P* = 0.0233; SYP +30.8%, *F*_4.75_= 40.53, *P* = 5.96 × 10^–5^; PSD95 +112.5%, *F*_4.75_= 61.06, *P* = 0.0018; GluA1 +25.4%, *F*_4.75_= 6.24, *P* = 0.0364; Fig. 7e and f). Finally, we repeated the treatment of sAD-N with either hAMSC-EVs or hAMSC-EV_R_ and analyzed the expression of SYN-1, SYP, PSD95, and GluA1. hAMSC-EV_R_ showed lower capability to prevent alterations of these proteins in sAD-N compared to naïve hAMSC-EVs (SYN-1 *P* = 0.0126; PSD95 *P* = 0.0021; GluA1 *P* = 0.0006; Fig. 7g, h). In summary, RNA-based cargoes of hAMSC-EVs counteract the altered expression of neuroplasticity-related proteins in sAD-N. hAMSC-EVs also prevent the AD-related changes of neuronal morphology, potentially exerting their beneficial effects by sustaining neuronal integrity.

## Discussion

AD is the leading cause of age-related dementia [37, 38]. It also represents a socio-economic emergency for the world health system due to its impact on the lives of both patients and their caregivers, and the lack of effective therapies [39]. Epidemiological evidence indicates that patients with chronic inflammatory conditions show a higher risk of developing neurodegenerative diseases [40]. Accordingly, *APOE* and *TREM2* variants, which have been established as the strongest genetic risk factors for the late-onset AD, promote neuroinflammation and exacerbate neuronal death [41]. Recently, MSC-derived EVs have emerged as a promising therapeutic agent for neurological disorders [16]. In this study, we found that multiple intranasal administration of hAMSC-EVs counteracted hippocampal neuroinflammation and the onset of cognitive deficits by modulating glial activation, microglial morphology, Aβ accumulation and synaptic protein expression in the hippocampus of female 3 × Tg-AD mice. These results suggest that hAMSC-EVs may engage endogenous neuroprotective pathways within the central nervous system. hAMSCs are well characterized for their potent immunomodulatory capacity, largely mediated through their secretome. We previously demonstrated that this immunoregulatory effect is predominantly due to soluble components, which suppress T-cell proliferation and differentiation toward inflammatory CD4 Th1 and CD8 cytotoxic subsets [42–44], skew monocyte polarization toward M2-like phenotypes [43, 45], inhibit dendritic cell maturation [43, 46], and attenuate B-cell activation and differentiation into antibody-producing cells [47]. These immunomodulatory properties have translated into therapeutic effects in vivo. In the R6/2 mouse model of Huntington’s disease, treatment with CM-hAMSC delayed the onset of motor dysfunction, improved behavioral outcomes, and reduced striatal pathology. These effects were accompanied by diminished microglial activation and inducible nitric oxide synthase expression, suggesting microglial modulation as a key mechanism [36]. Similarly, in models of traumatic brain injury, hAMSC or CM-hAMSC administration provided early and sustained neuroprotection, including neuronal rescue and the promotion of M2 microglial polarization, even in the absence of direct tissue contact [48].

Neuroinflammation is now widely recognized as a critical factor that exacerbates the progression of many neurodegenerative disorders, including AD. This inflammatory state compromises neuronal health and contributes to the overall decline in brain function [11]. Moreover, MSC-derived EVs have been reported to exert anti-inflammatory effects and counteract the nervous system degeneration in multiple neurodegenerative disorders [49, 50]. A detailed miRNA profiling of hAMSC-EVs revealed an enrichment of teno- and chondro-protective miRNAs, capable of modulating macrophage polarization, reducing T cell inflammatory responses, and promoting Treg expansion [19]. Our data in the 3 × Tg-AD model further support this interpretation. hAMSC-EV administration resulted in a significant reduction in Iba1⁺ microglial and GFAP⁺ astrocytic density in the hippocampus and entorhinal cortex (Fig. 3). Microglia displayed a shift from hyper-ramified, pro-inflammatory morphology to simplified ameboid or moderately ramified phenotypes, consistent with a transition toward less reactive states [51, 52]. These morphological changes were paralleled by reduced expression of TMEM119 and TREM2, markers of disease-associated microglia (DAM). TMEM119 downregulation reflects a transition away from DAM status and is typically observed near neurodegenerative lesions [53]. TREM2, a key regulator of microglial metabolism and phagocytosis, is essential for DAM progression. TREM2 is often considered neuroprotective because its lack may accelerate neuronal loss and progression of neuroinflammation [54]. However, its genetic deficiency in AD models has been associated with reduced IL-1β and IL-6 levels and increased expression of anti-inflammatory mediators such as Ym1 and Fizz1 [55]. Our data suggest that hAMSC-EVs may inhibit the transition to a fully activated DAM state, reduce Aβ phagocytosis and promote a homeostatic microglial phenotype. Microglia play a complex, multifaceted role in AD. Although they act as neuroprotectors by phagocytosing pathological Aβ and neuronal debris in early stages, as the disease progresses microglia become hyperactive, leading to toxic and pro-inflammatory state. Restoring a more homeostatic, anti-inflammatory state and downregulating microglial Aβ uptake can counteract AD progression by preventing the formation of highly destructive "dense-core" plaques and quenching chronic neuroinflammation [56]. Accordingly, the reduced microglial Aβ uptake observed in vitro (Fig. 6a) may reflect a tolerant, homeostatic microglial state rather than impaired clearance [57, 58]. The net decrease of hippocampal Aβ, occurring without changes in BACE1, IDE or Neprilysin (Fig. S1a), may stem mainly from reductions of inflammation-driven amyloid generation and dense-core plaque compaction rather than enhanced enzymatic degradation, in line with the non-phlogistic Aβ clearance reported upon dampening of innate immune signaling, such as NLRP3 inhibition [59]. In addition, the abundance of immunoregulatory cytokines such as IL-6, IL-13, and IL-4 within the EV cargo mirrors the anti-inflammatory shift observed in the hippocampus, suggesting that hAMSC-EVs may directly transfer these bioactive factors to the brain microenvironment. Notably, in many studies, IL-6 is considered a proinflammatory cytokine enhancing neuroinflammation and promoting cognitive decline in AD [60, 61]. However, similar to microglial activation, IL-6 may exhibit context-dependent ambivalent roles in AD. Beyond the neurotoxic effects, IL-6 can exert a neuroprotective role by increasing BDNF release [62], regulating brain glucose metabolism [63], preserving blood–brain barrier integrity [64], and promoting microglia-dependent Aβ phagocytosis [59], brain repair and synaptic plasticity [65]. Finally, Mendelian randomization studies demonstrated that high levels of IL-6 are significantly associated with reduced risk of AD [66, 67].

Comprehensive profiling of the miRNA cargo of hAMSC-EVs revealed a coordinated regulatory network relevant to AD. Several upregulated miRNAs (including miR-146a-5p, miR-483-5p and miR-148a-3p) showed strong AD associations in the HMDD database. GO and KEGG analyses indicated enrichment in pathways linked to nervous system development, immune regulation, neurodegeneration, and aging. Predicted targets of the up- and downregulated miRNAs showed distinct functional patterns: targets of the downregulated miRNAs were primarily associated with immune regulation and neural development, whereas targets of the upregulated miRNAs were enriched for neurogenesis, axon guidance, and neuronal differentiation. Several biological processes (including axogenesis and dendrite development) were influenced by both groups.

Analysis of the murine orthologs further supported conserved neuroprotective functions. miR-146a-5p and miR-410-3p, which are enriched in the hAMSC-EV cargo, have murine orthologs with identical seed sequences, and the predicted targets of mmu-miR-146a-5p and mmu-miR-410-3p include microglial markers such as TMEM119 and TREM2. The processes enriched among these predicted targets relate to immune modulation, glial differentiation and axon guidance, reinforcing the potential involvement of this cargo in AD-relevant signaling. Among the EV miRNAs, miR-146a-5p emerged as a key regulator of microglial activation and neuroinflammation, while miR-142a-5p contributed to neuronal survival and synaptic restoration through modulation of BAI3, GSK-3β, and CDK5-related pathways. Additional EV-enriched miRNAs, including miR-132/212, miR-483-5p, and miR-148a-3p, collectively target tau phosphorylation, amyloid processing, inflammatory signaling, and memory-related pathways. Importantly, because our strategy degraded the bulk of vesicular RNA rather than a single species, these experiments establish that the EV RNA cargo as a whole, rather than any individual transcript, is causally required for the anti-inflammatory activity of hAMSC-EVs. Among the co-depleted inflammation-related miRNAs, miR-146a-5p is a plausible candidate effector, as it restrains the TLR4/IRAK1/TRAF6/NF-kB axis [57] and, when enriched in MSC-derived EVs, skews microglia toward an anti-inflammatory phenotype in a manner reverted by its selective knockdown [68]. This is consistent with the reduced NF-kB phosphorylation and increased CD206 detected in the LPS-stimulated microglia (Fig. 6c). More broadly, the EV cargo might promote innate immune tolerance rather than innate immune training (i.e., the pro-inflammatory memory phenotype of primed microglia) in microglia, an imprinting reported to alleviate β-amyloidosis [58]. Our findings also showed no effects of hAMSC-EVs on tau activation. Several studies have indicated that therapeutic interventions may selectively target either Aβ or tau pathology, reducing Aβ burden without affecting tau hyperphosphorylation, or vice versa [69–71]. Consistent with this notion, intravenous administration of MSCs was reported to attenuate tau phosphorylation in 3 × Tg-AD mice without altering Aβ levels [72], suggesting that components of the stem cell secretome may differentially modulate Aβ- and tau-related pathogenic pathways. However, RNA depletion experiments highlighted the role of RNA-based mechanisms (e.g., miR-125b-5p, miR-146a-5p, and miR-423-5p) in the anti-inflammatory and pro-neuroplasticity-related effects of hAMSC-EVs. Together, these findings indicate that hAMSC-EVs deliver a combination of miRNAs capable of modulating key pathological processes in AD, including microglial activation, amyloid processing, synaptic dysfunction, neuroinflammation, and cognitive impairment. The rescue of synaptic protein expression in both the hippocampus of 3 × Tg-AD mice and human neurons derived from AD patients and, more importantly, the beneficial effects on neuronal integrity of sAD-hN, add new insights into the translational potential of hAMSC-EVs. The context-dependent and multifactorial actions of these miRNAs underscore the therapeutic potential of hAMSC-EVs as a non-cellular, multi-targeted approach to modulate neurodegeneration. However, further in vivo validation is necessary to fully delineate the contribution of individual miRNAs and to optimize EV-based strategies for translational application in AD.

## Conclusions

Collectively, our findings indicate that hAMSC-EVs attenuate neuroinflammation by reshaping the inflammatory microenvironment rather than through direct immunosuppression. The observed changes in microglial phenotype, cytokine profile, and neuroplasticity-related factors suggest that hAMSC-EVs promote a permissive environment that supports neuronal integrity, synaptic function, and the homeostasis of other brain cell populations. These mechanisms are particularly relevant in AD, where chronic inflammation, synaptic dysfunction, and neurodegeneration are tightly interconnected. By influencing multiple pathological processes simultaneously, hAMSC-EVs reinforce their potential as a cell-free therapeutic strategy for neurodegenerative disorders [73].

Although preliminary evidence suggests that hAMSC-EVs may also attenuate cognitive decline at advanced stages of disease, further studies are required to optimize treatment protocols, define the molecular mechanisms and miRNA signaling networks responsible for their neuroprotective effects, and improve brain-targeted delivery. In this context, advances in EV engineering, including surface modification and cargo optimization, may further enhance therapeutic specificity, bioavailability, and translational potential for AD and other neurodegenerative disorders [74, 75].

## Supplementary Information

Additional file 1. **Fig. S1**. hAMSC-EV treatment partially exerts therapeutic effects. **Fig. S2**. Characterization of healthy CTR and AD patients-derived hiPSCs. **Fig. S3**. RNA depletion protocol does not alter EV integrity. **Fig. S4**. Uncropped blots. **Table S1**. Primary antibodies. **Table S2**. *P*-value and fold regulation of inflammation-related miRNAs.

## Data Availability

The datasets used and/or analyzed during the current study are available from the corresponding author on reasonable request.

## Abbreviations

AD: Alzheimer’s disease
Aβ: Amyloid-β
BP: Biological process
DIV: Days in vitro
EV: Extracellular vesicles
GO: Gene Onthology
hiPSCs: Human induced pluripotent stem cells
hNs: Human neurons
miRNAs: MicroRNAs
MSCs: Mesenchymal stromal cells
NFTs: Neurofibrillary tangles
NOR: Novel object recognition test
OPR: Object place recognition test
sAD: Sporadic Alzheimer’s disease
VEH: Vehicle
