# Supplementary material for: Human amniotic mesenchymal stromal cell-derived extracellular vesicles reprogram microglia and prevent neurodegeneration in experimental models of Alzheimer’s disease
Source: Transl Neurodegener. 2026 Sep 9;15:46. doi: 10.1186/s40035-026-00581-1 (PMC13556012; doi:10.1186/s40035-026-00581-1)
Supplement: Supplementary file 1 [file 40035_2026_581_MOESM1_ESM.pdf]

Human amniotic mesenchymal stromal cell-derived extracellular vesicles reprogram microglia and prevent neurodegeneration in experimental models of Alzheimer's disease

Andrea Papait[1,2]*, Francesca Natale[2,3]*, Antonietta Rosa Silini[4], Ida Nifo Sarrapochiello[2], Elisa Orecchini[1,5], Raimondo Sollazzo[2,3], Marco Rinaudo[2,3], Serafina Farigu[4], Nicoletta Garofalo[3], Giulia Mantini[5], Guido Maria Giuffrè[2,3], Pietro Romele[4], Elvira Ragozzino[1], Alice Dellaria[3], Luciano Giacò[5], Camillo Marra[2,6], Cristian Ripoli[2,3], Claudio Grassi[2,3], Ornella Parolini[1,7#], Salvatore Fusco[2,3#]

[1] Dipartimento di Scienze della Vita e Sanità Pubblica, Università Cattolica del Sacro Cuore, 00168 Roma, Italy

[2] Fondazione Policlinico Universitario A. Gemelli IRCCS, 00168 Roma, Italy

[3] Dipartimento di Neuroscienze, Università Cattolica del Sacro Cuore, 00168 Roma, Italy

[4] Centro di Ricerca E. Menni, Fondazione Poliambulanza Istituto Ospedaliero, 25124 Brescia, Italy

[5] UOS Computational Biology and Bioinformatics GSTeP, Fondazione Policlinico Universitario A: Gemelli IRCCS, 00168 Roma, Italy

[6] Dipartimento di Psicologia, Università Cattolica del Sacro Cuore, 20123 Milano, Italy

[7] Fondazione IRCCS Casa Sollievo della Sofferenza, San Giovanni Rotondo, 71013 Foggia, Italy

Andrea Papait andrea.papait@unicatt.it

Francesca Natale francesca.natale1@unicatt.it

Antonietta Rosa Silini antonietta.silini@poliambulanza.it

Ida Nifo Sarrapochiello ida.nifosarrapochiello@unicatt.it

Elisa Orecchini elisa.orecchini@unicatt.it

Raimondo Sollazzo raimondo.sollazzo@unicatt.it

Marco Rinaudo marco.rinaudo@unicatt.it

Serafina Farigu serafina.farigu@poliambulanza.it

Nicoletta Garofalo nicoletta.garofalo@unicatt.it

Giulia Mantini giulia.mantini@policlinicogemelli.it

Guido Maria Giuffrè guidomaria.giuffre@policlinicogemelli.it

Pietro Romele pietro.romele@poliambulanza.it

Elvira Ragozzino elvira.ragozzino@unicatt.it

Alice Dellaria alice.dellaria@unicatt.it

Luciano Giacò luciano.giaco@policlinicogemelli.it

Camillo Marra camillo.marra@unicatt.it

Cristian Ripoli cristian.ripoli@unicatt.it

Claudio Grassi claudio.grassi@unicatt.it

Ornella Parolini ornella.parolini@unicatt.it

Salvatore Fusco salvatore.fusco@unicatt.it

Correspondence: claudio.grassi@unicatt.it

* These authors share first authorship

# These authors share last authorship

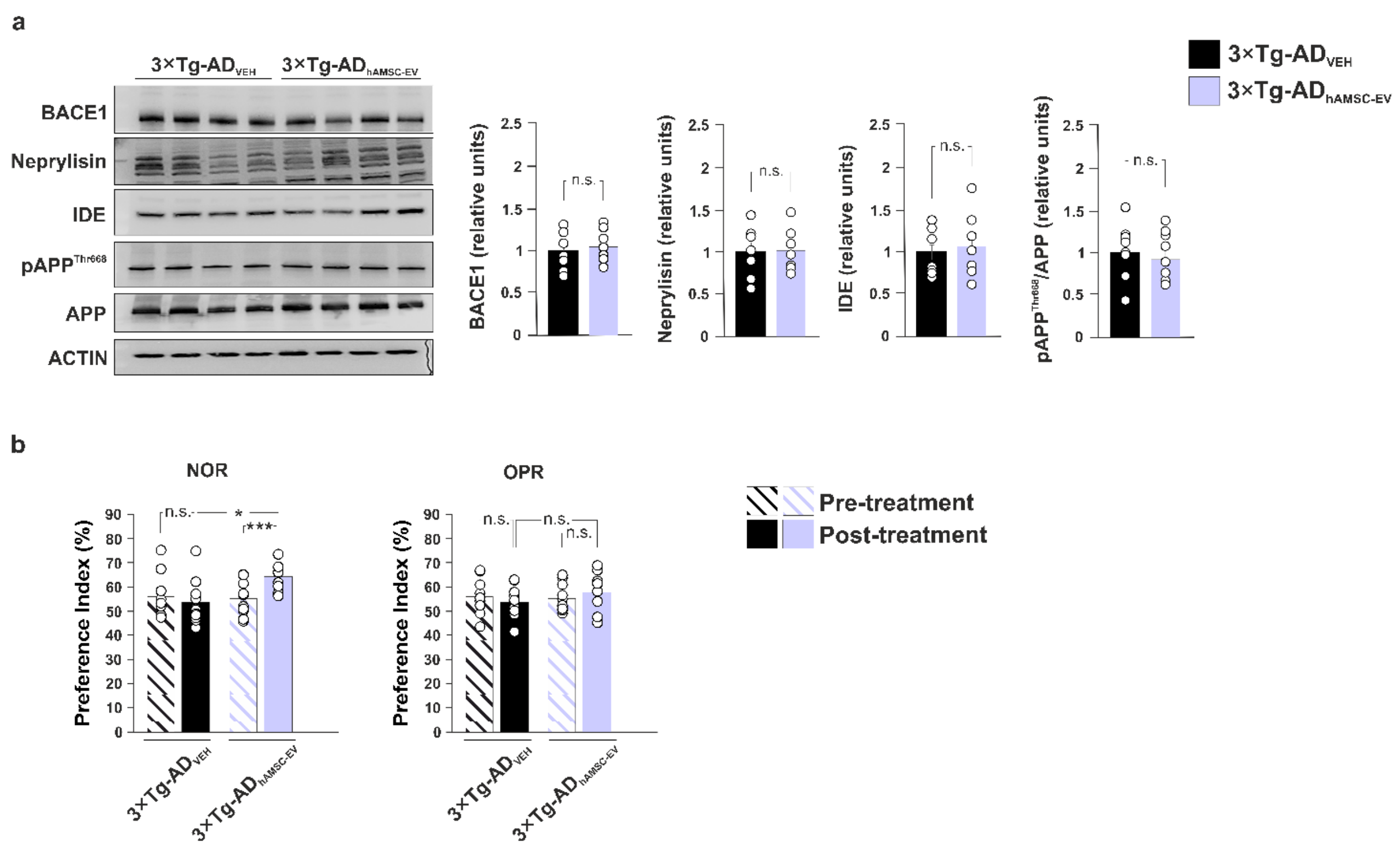

Figure S1. hAMSC-EVs treatment partially exerts therapeutic effects. a Immunoblots (left) and bar graphs (right) showing the expression levels of IDE, neprylisin, BACE1, and phospo-APP in the hippocampus of both 3×Tg-AD$_{VEH}$ and 3×Tg-AD$_{hAMSC-EV}$ mice (n=8 mice per group; statistics by unpaired Student's t-test). **b** Preference indexes for the NOR (left) and OPR (right) tests of 9-month-old 3×Tg-AD female mice treated for 1 month with vehicle or

hAMSC-EV (n=10 per group; PI NOR: 55.89% pre- and 53.48% post-treatment for 3×Tg-AD_{VEH} , 55.23% pre- and 64.34% post-treatment for 3×Tg-$AD_{hAMSC-EV}$, P = 0.0109 for post-treatment PIs; PI OPR: 56.16% pre- and 53.94% post-treatment for 3×Tg-AD_{VEH} , 55.71% pre- and 58.24% post-treatment for 3×Tg-$AD_{hAMSC-EV}$, P = 0.3322; statistics by 2-way ANOVA for repeated measures and Sidak post-hoc test). Data are expressed as mean ± SEM. *$p < 0.05$; ***$p < 0.001$; n.s. not significant.

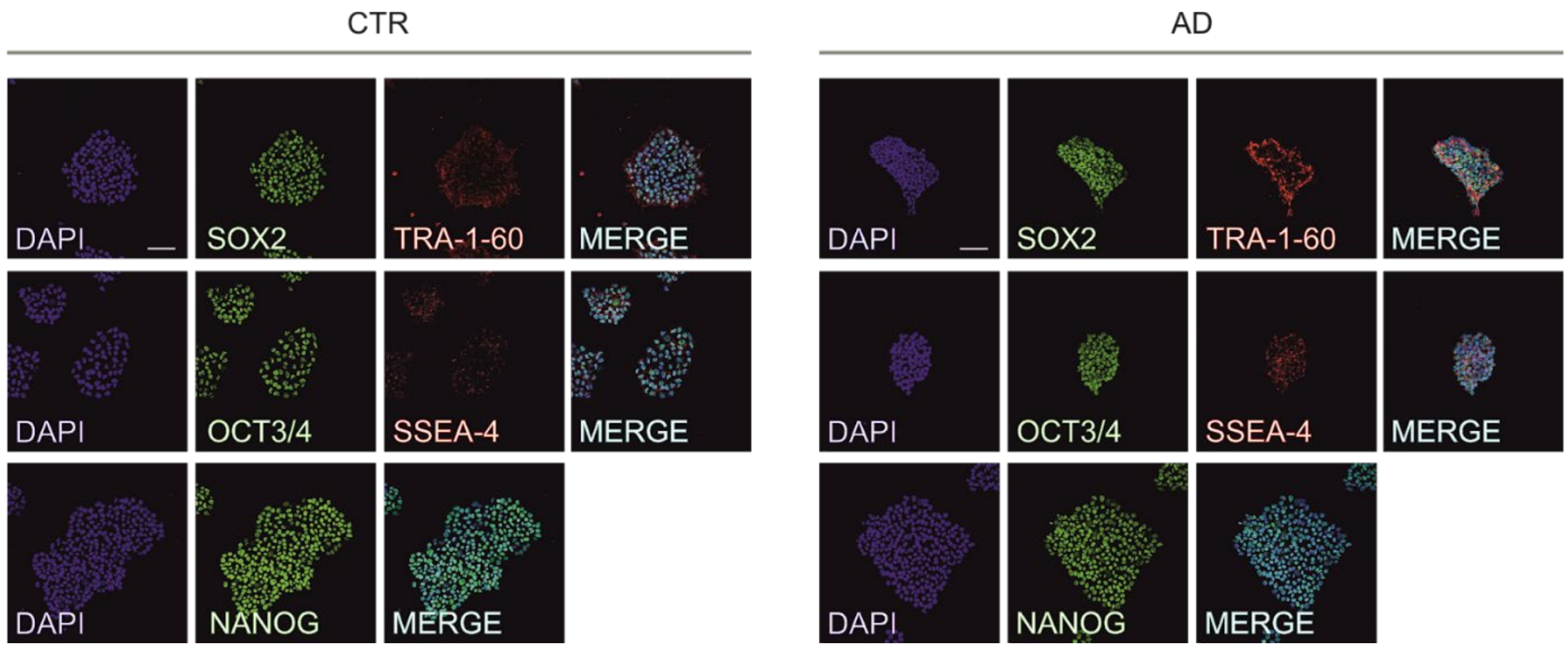

Figure S2. **Characterization of healthy CTR and AD patients-derived hiPSCs.** Representative immunofluorescence images showing the expression of pluripotency markers in hiPSCs derived from healthy subjects (CTR) and sporadic Alzheimer's disease (AD) patients. Cells were stained for SOX2 (green), NANOG (green), OCT3/4 (green), SSEA-4 (red), and TRA-1–60 (red), with nuclei counterstained using DAPI (blue). hiPSC lines from both CTR and AD donors displayed robust expression of pluripotency-associated markers, confirming their pluripotent stem cell identity. Scale bar: 50 µm.

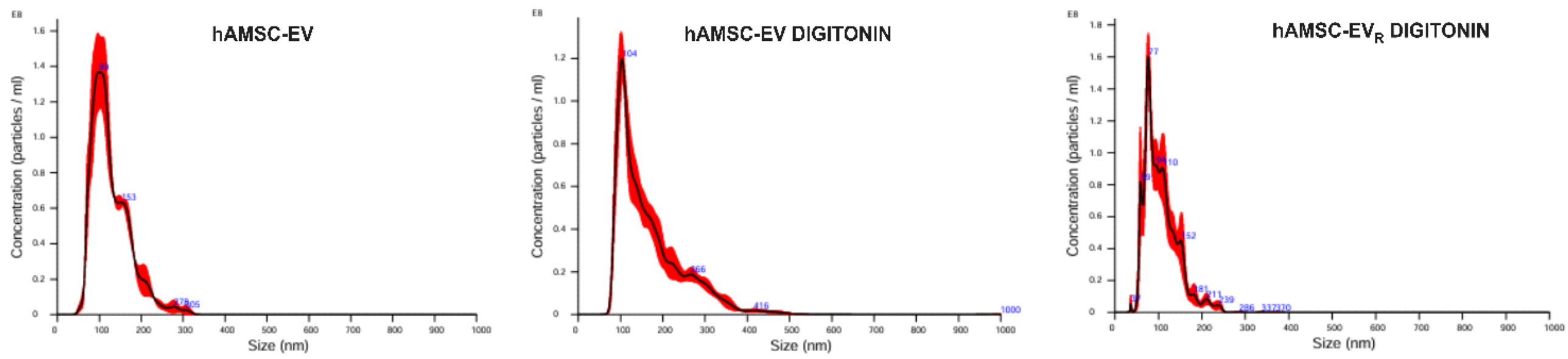

Figure S3. RNA depletion protocol does not alter EV integrity. NTA analysis of untreated hAMSC-EVs (left), digitonin-treated hAMSC-EVs (centre) and digitonin/RNAse-treated hAMSC-EVs (right).

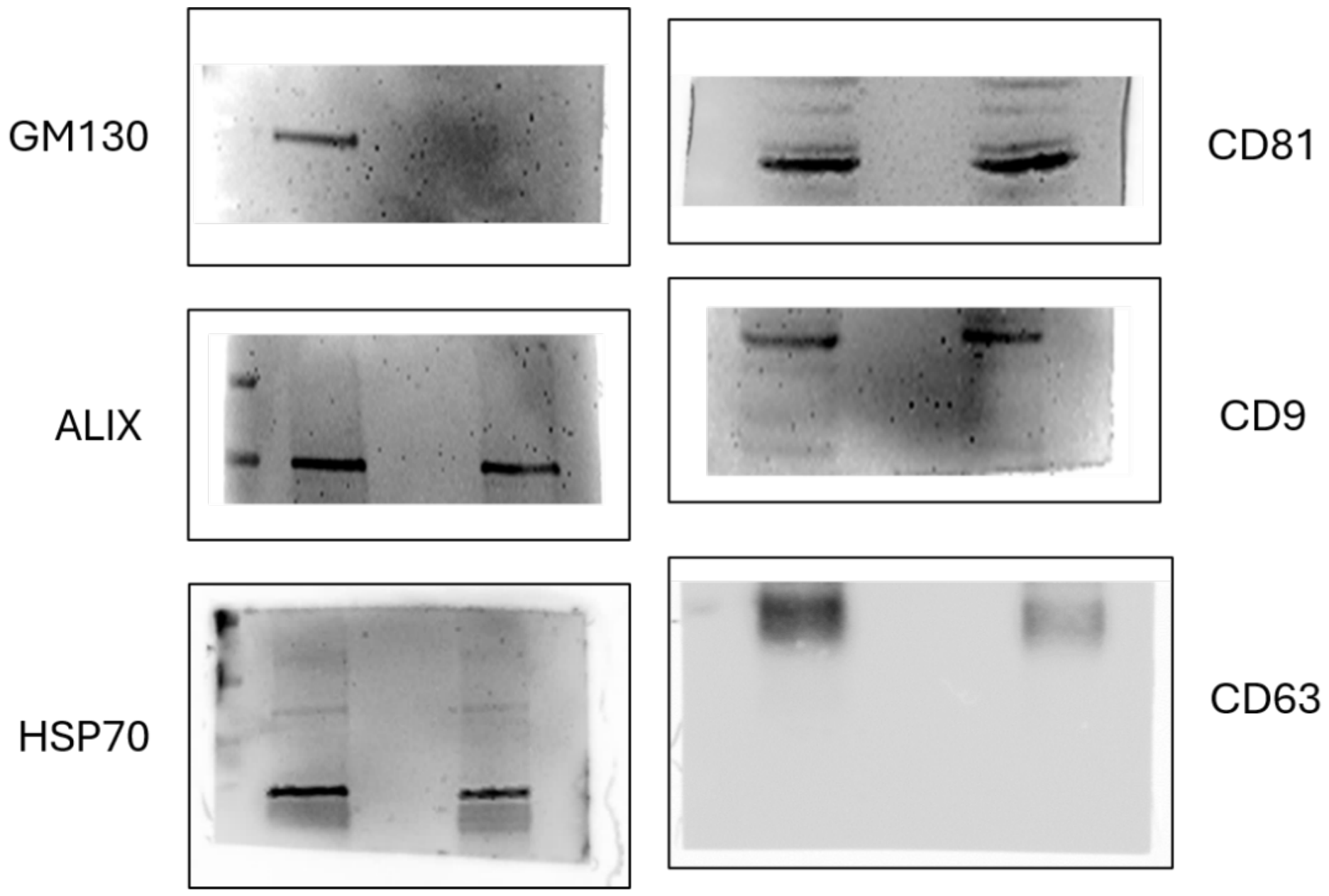

Uncropped Blots Fig. 1b.

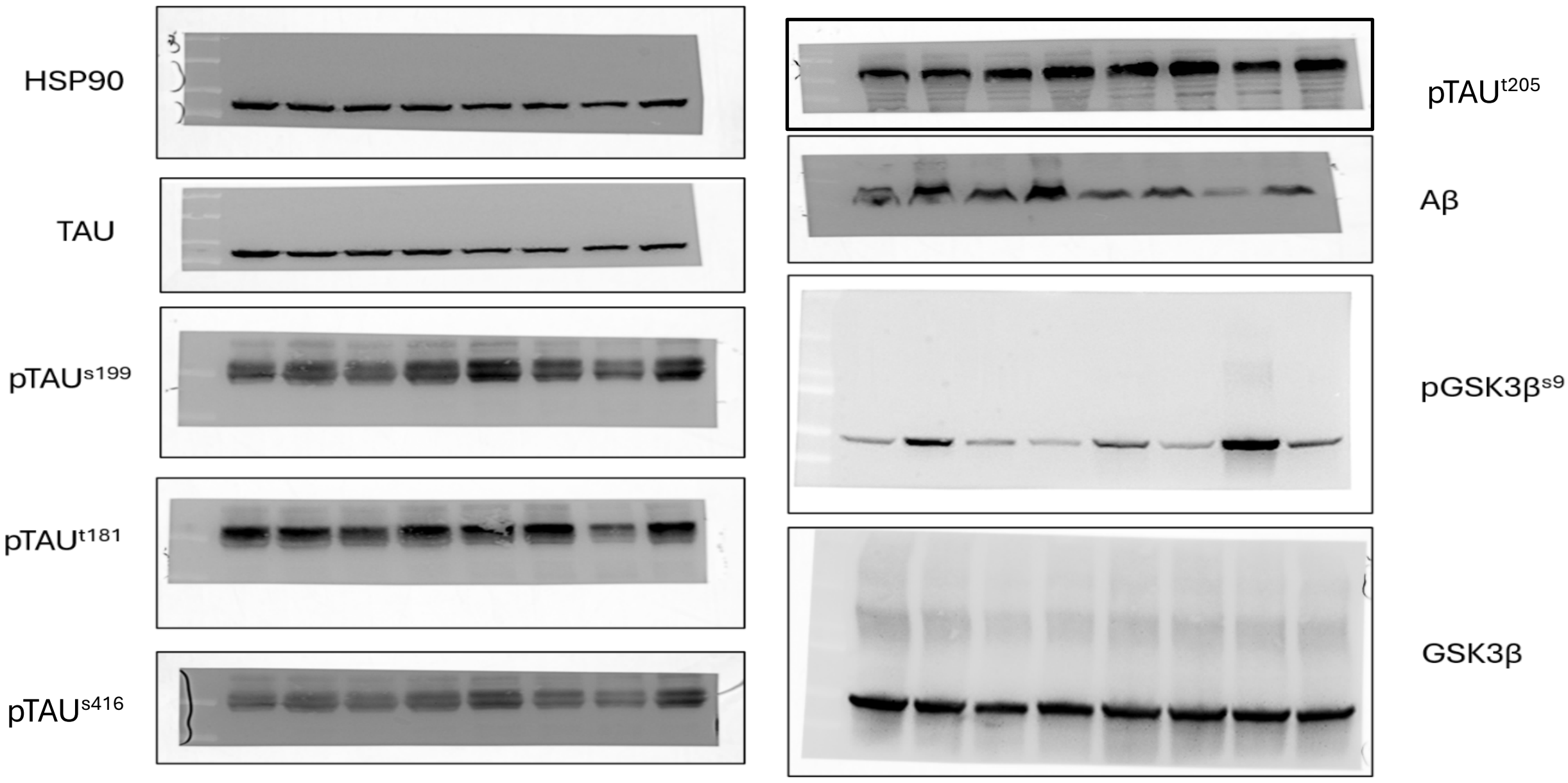

Uncropped Blots Fig. 2e.

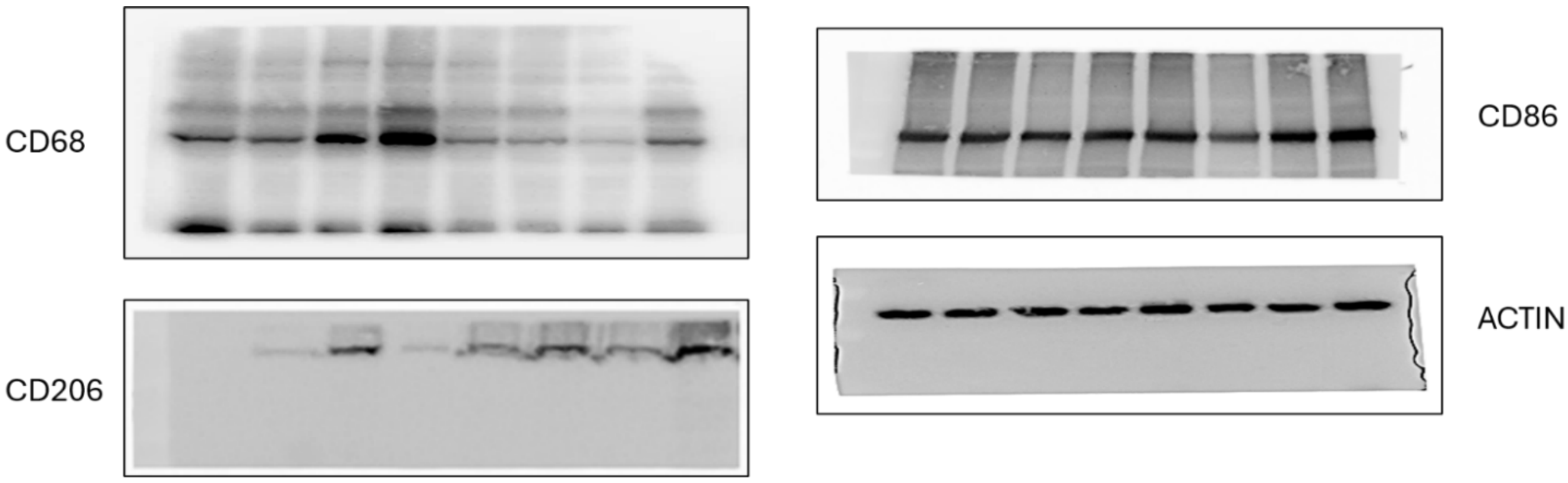

Uncropped Blots Fig. 2g.

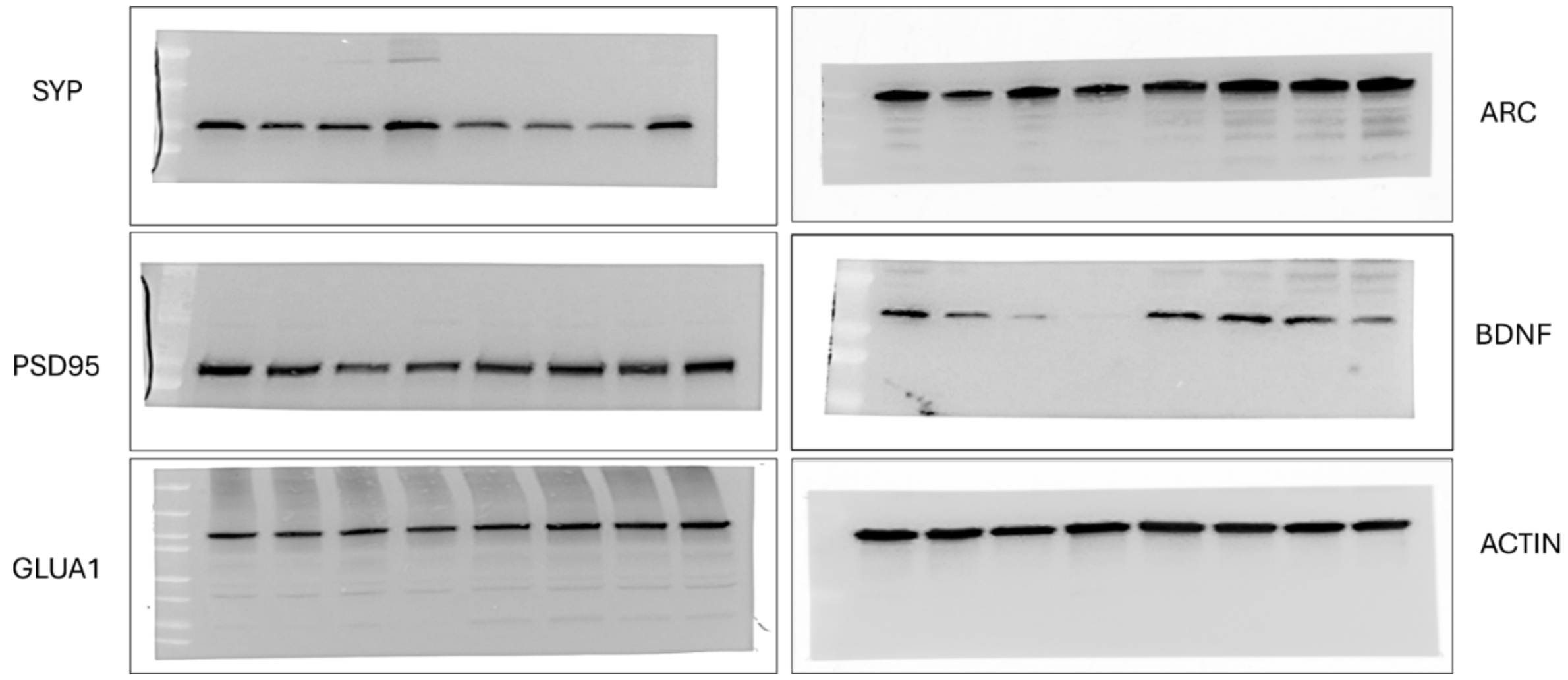

Uncropped Blots Fig. 5d.

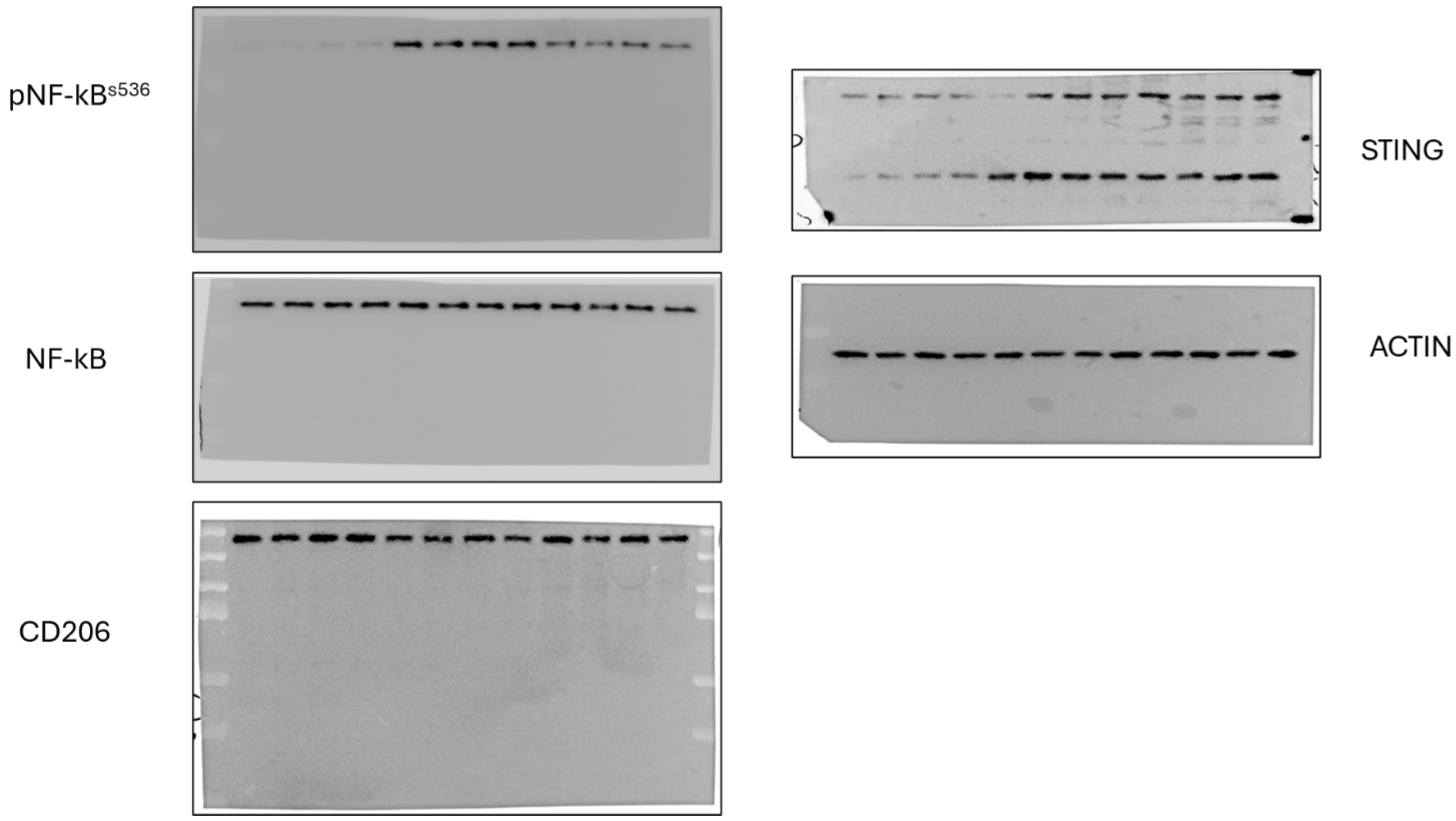

Uncropped Blots Fig. 6c.

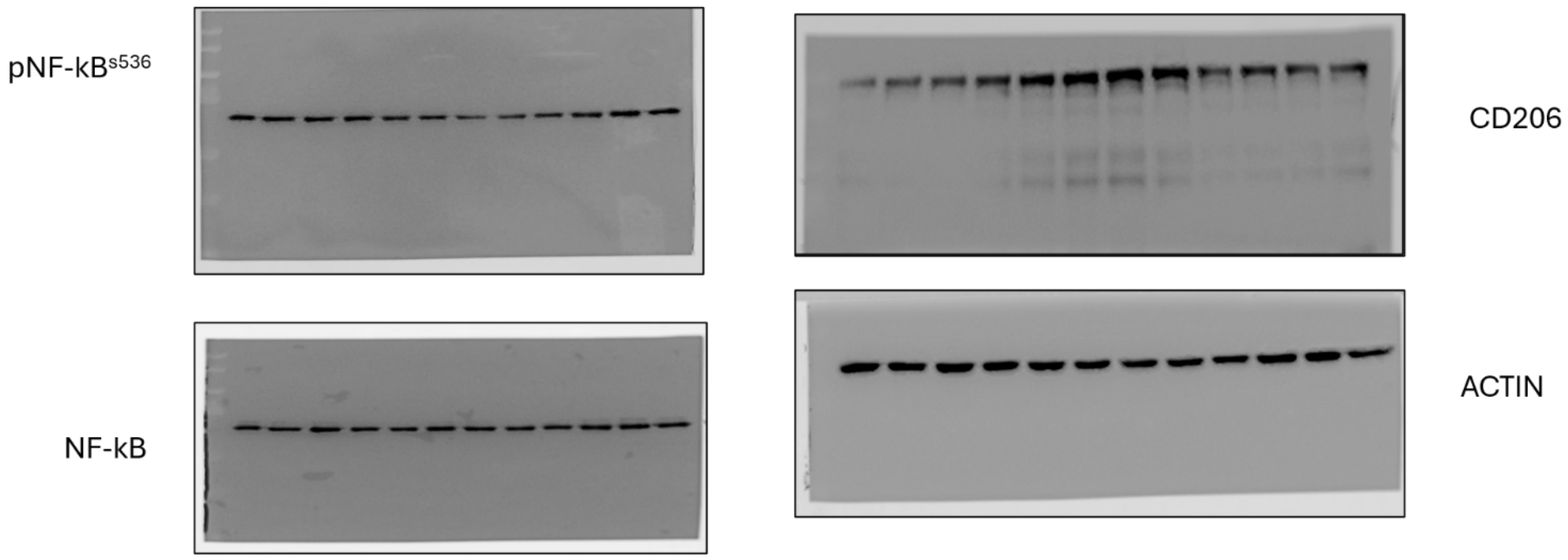

Uncropped Blots Fig. 6d.

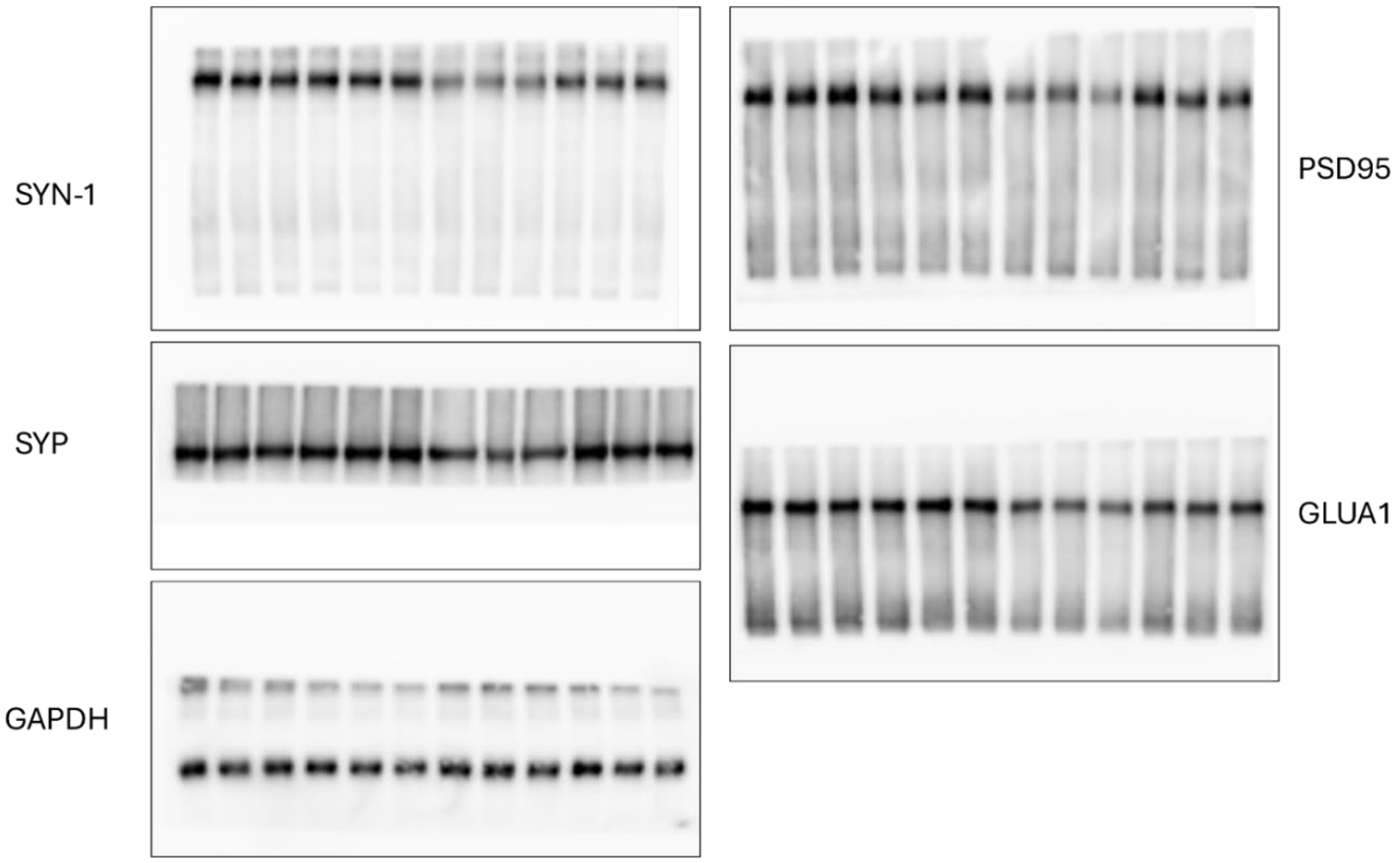

Uncropped Blots Fig. 7e.

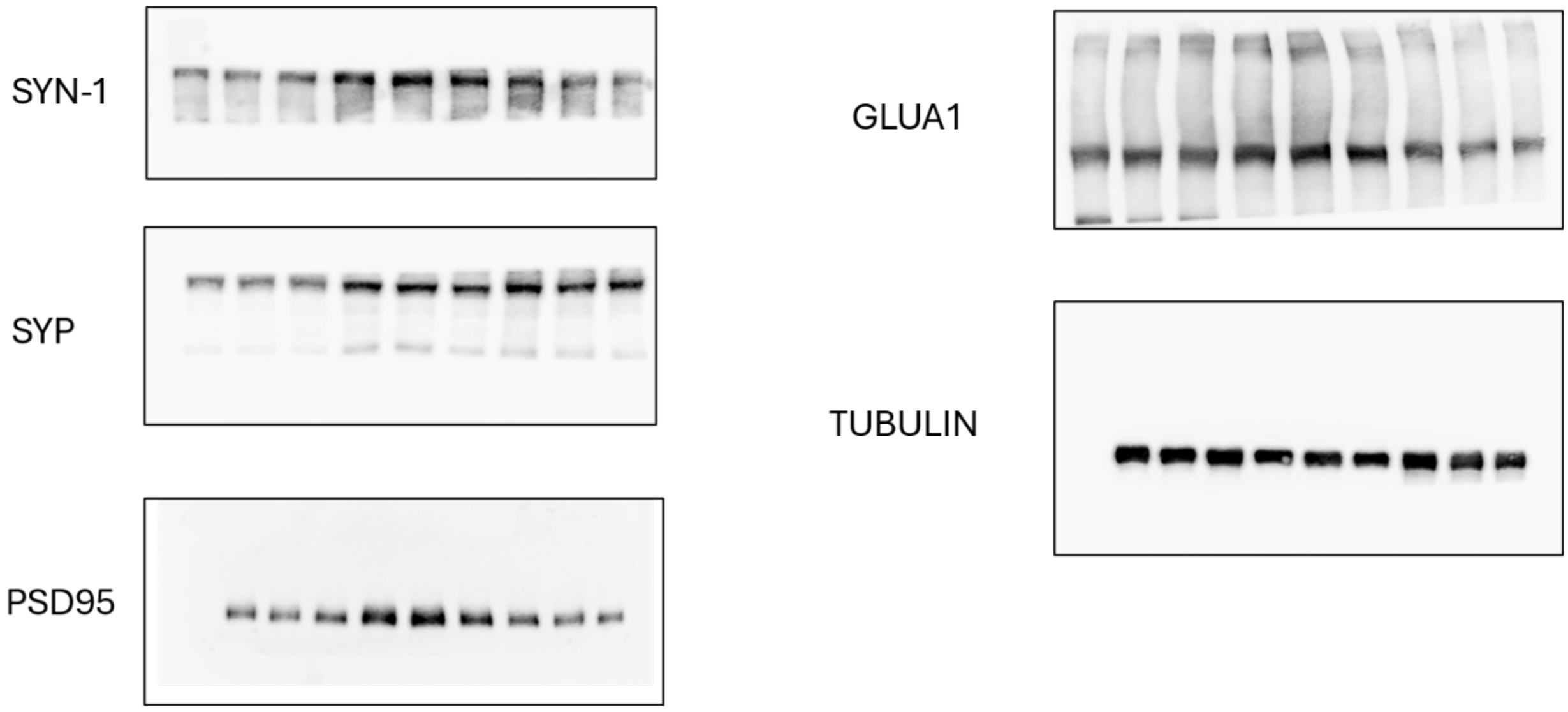

Uncropped Blots Fig. 7g.

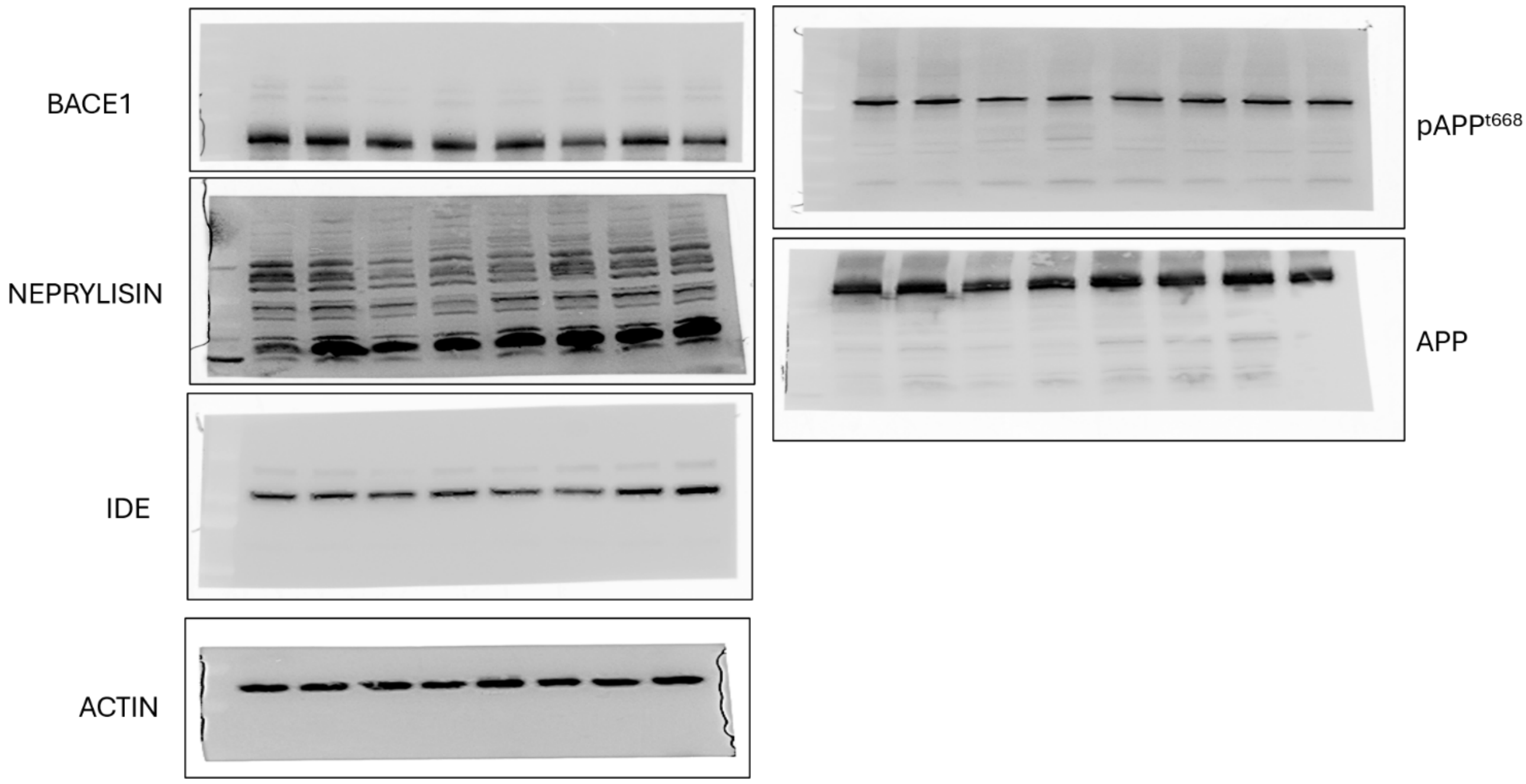

Uncropped Blots Fig. S1a.

Figure S4. Uncropped Blots.

Table S1. Primary Antibodies.

Antibody	Host & Dilution	Cat. Reference	Exp.
**α-β-Amyloid (D54D2)**	Rabbit 1:1000	Cell Signaling Technology #8243	WB
**α-TMEM119**	Rabbit 1:250	Genetex AB_2887209	IF
**α-TREM2**	Goat 1:50	Abcam ab95470	IF
**α-GFAP**	Chicken 1:1000	Genetex AB_10621124	IF
**α-IBA-1**	Chicken 1:1000	Invitrogen AB_2942801	IF
**α-IBA-1**	Rabbit 1:500	Immunological Sciences AB-83747	IF
**TAU (D1M9X) XP 46687S**	Rabbit 1:1000	Cell Signaling Technology #46687S	WB
**pTAU (S416) (D7U2P) 15013T**	Rabbit 1:1000	Cell Signaling Technology #15013T	WB
**pTAU (T205) (E703E)**	Rabbit 1:1000	Cell Signaling Technology #49561S	WB
**pTAU (T181) (D9F44)**	Rabbit 1:1000	Cell Signaling Technology #12885S	WB
**pTAU (S199)**	Rabbit 1:1000	Cell Signaling Technology #29957S	WB
**α-SYNAPTOPHYSIN**	Mouse 1:1000	Abcam ab8049	WB
**α-PSD95 (D27E11) XP**	Rabbit 1:1000	Cell Signaling Technology #3450S	WB
**α-GLUA1 (D4N9V)**	Rabbit 1:1000	Cell Signaling Technology #13185S	WB
**α-ARC**	Rabbit 1:1000	Abcam ab183183	WB
**α-GM 130**	Mouse 1:1000	BD Biosciences 610822	WB
**α-ALIX (3A9)**	Mouse 1:1000	Cell Signaling Technology #2171	WB
**α-HSP70**	Rabbit 1:1000	Cell Signaling Technology #4872	WB
**α-CD81 (D5O2Q)**	Rabbit 1:1000	Cell Signaling Technology #10037	WB
**α-CD9**	Rabbit 1:1000	Abcam ab92726	WB
**α-BDNF**	Rabbit 1:1000	Cell Signaling Technology #47808S	WB

**α-ACTIN (8H10D10)**	Mouse 1:1000	Cell Signaling Technology #3700	WB
**α-SYNAPSIN-1**	Rabbit 1:1000	Cell Signaling Technology #5297	WB
**α-GAPDH**	Mouse 1:1000	Abcam ab8245	WB
**α-HSP90**	Rabbit 1:1000	Cell Signaling Technology #4877S	WB
**MAP2 (D5G1)**	Rabbit 1:500	Cell Signaling Technology #8707	IF
**F-Actin (Alexa Fluor^{TM} 546)**	1:500	Thermo Fisher Scientific R415	IF
**pAPP**	Rabbit 1:1000	Cell Signaling Technology #6986	WB
**APP**	Rabbit 1:1000	Abcam ab32136	WB
**BACE1 (D10ES)**	Rabbit 1:1000	Cell Signaling Technology #5606	WB
**CD10/NEPRYLISIN (F4P6H)**	Rabbit 1:1000	Cell Signaling Technology #58145	WB
**IDE**	Rabbit 1:1000	Abcam ab32216	WB
**pGSK3β (D85E12)**	Rabbit 1:1000	Cell Signaling Technology #5558	WB
**GSK3β (3D10)**	Rabbit 1:1000	Cell Signaling Technology #9832	WB
**CD68 (E307W)**	Rabbit 1:1000	Cell Signaling Technology #97778	WB
**CD86**	Rabbit 1:1000	Immunological Sciences AB84415	WB
**CD206/MRC1**	Rabbit 1:1000	Cell Signaling Technology #24595	WB
**pNFKB p65**	Rabbit 1:1000	Abcam ab76302	WB
**NFKB p65**	Rabbit 1:1000	Abcam ab16502	WB
**STING(D2P2F)**	Rabbit 1:1000	Cell Signaling Technology #13647S	WB
**CD63**	Rabbit 1:1000	Abcam AB217345	WB

WB: Western Blot Experiments; IF: Immunofluorescence Experiments

Table S2. p-value and Fold regulation of inflammation -related-miRNAs

miRNA	p-value	Fold Regulation
hsa-let-7a-5p	0.347015	-3.86
hsa-let-7b-5p	**0.036671**	**-11.26**
hsa-let-7c-5p	Undetermined	N/A
hsa-let-7d-5p	Undetermined	N/A
hsa-let-7e-5p	0.060570	-20.04
hsa-let-7f-5p	Undetermined	NA
hsa-let-7g-5p	Undetermined	NA
hsa-let-7i-5p	**0.000485**	**-48.78**
hsa-miR-100-5p	Undetermined	NA
hsa-miR-101-3p	Undetermined	NA
hsa-miR-106b-5p	0.210935	-10.82
hsa-miR-125b-5p	**0.000559**	**-5558.96**
hsa-miR-126-3p	Undetermined	NA
hsa-miR-128-3p	Undetermined	NA
hsa-miR-130b-3p	Undetermined	NA
hsa-miR-132-3p	**0.036710**	**-14.69**
hsa-miR-139-5p	Undetermined	NA
hsa-miR-142-3p	Undetermined	NA
hsa-miR-142-5p	Undetermined	NA
hsa-miR-145-5p	0.100390	-27.45
hsa-miR-146a-5p	**0.014335**	**-1196.45**
hsa-miR-146b-5p	Undetermined	NA
hsa-miR-147a	0.180050	-2.95
hsa-miR-148a-3p	Undetermined	NA
hsa-miR-150-5p	0.075178	-14.61
hsa-miR-155-5p	Undetermined	NA
hsa-miR-15a-5p	Undetermined	NA
hsa-miR-15a-3p	Undetermined	NA
hsa-miR-15b-5p	Undetermined	NA
hsa-miR-16-5p	Undetermined	NA
hsa-miR-17-5p	Undetermined	NA
hsa-miR-17-3p	0.116236	-6.01

hsa-miR-181a-5p	**0.000821**	**-260.73**
hsa-miR-181b-5p	0.073730	-10.21
hsa-miR-181c-5p	Undetermined	NA
hsa-miR-181d-5p	Undetermined	NA
hsa-miR-182-5p	Undetermined	NA
hsa-miR-184	Undetermined	NA
hsa-miR-18a-5p	Undetermined	NA
hsa-miR-191-5p	**0.046646**	**-22.59**
hsa-miR-195-5p	Undetermined	NA
hsa-miR-199a-5p	Undetermined	NA
hsa-miR-19a-3p	0.282598	-4.36
hsa-miR-19b-3p	**0.020746**	**-97.30**
hsa-miR-204-5p	Undetermined	NA
hsa-miR-20a-5p	0.263099	-4.78
hsa-miR-20b-5p	Undetermined	NA
hsa-miR-21-5p	0.063817	-343.58
hsa-miR-210-3p	**0.003281**	**-23.64**
hsa-miR-214-3p	**0.023035**	**-162.79**
hsa-miR-221-3p	**0.033701**	**-392.85**
hsa-miR-222-3p	**0.028892**	**-98.94**
hsa-miR-223-3p	Undetermined	NA
hsa-miR-23a-3p	**0.004140**	**-186.54**
hsa-miR-23b-3p	Undetermined	NA
hsa-miR-24-3p	**0.000861**	**-118.73**
hsa-miR-25-3p	**0.002093**	**-127.84**
hsa-miR-26a-5p	Undetermined	NA
hsa-miR-26b-5p	Undetermined	NA
hsa-miR-27a-3p	**0.016972**	**-148.54**
hsa-miR-27b-3p	0.303644	-7.48
hsa-miR-28-5p	0.999633	-1.45
hsa-miR-29a-3p	**0.040060**	**-2.85**
hsa-miR-29b-3p	0.100782	-212.82
hsa-miR-29c-3p	0.109277	-18.64
hsa-miR-30a-5p	Undetermined	NA

hsa-miR-30b-5p	Undetermined	NA
hsa-miR-30c-5p	Undetermined	NA
hsa-miR-30d-5p	**0.004609**	**-26.92**
hsa-miR-30e-5p	Undetermined	NA
hsa-miR-31-5p	0.095962	**-16.68**
hsa-miR-326	0.052051	**-39.32**
hsa-miR-331-3p	Undetermined	NA
hsa-miR-335-5p	Undetermined	NA
hsa-miR-342-3p	0.100905	-33.30
hsa-miR-346	Undetermined	NA
hsa-miR-34a-5p	**0.000044**	**-215.13**
hsa-miR-365a-3p	0.094149	-75.05
hsa-miR-423-5p	**0.001896**	**-793.68**
hsa-miR-574-3p	**0.043461**	**-28.91**
hsa-miR-92a-3p	0.064505	-34.00
hsa-miR-93-5p	0.081196	-31.64
hsa-miR-98-5p	Undetermined	NA
hsa-miR-99a-5p	Undetermined	NA
